# Density Functional Theory Investigations of D-A-D' Structural Molecules as Donor Materials in Organic Solar Cell

**DOI:** 10.3389/fchem.2018.00200

**Published:** 2018-06-04

**Authors:** Junxian Chen, Qingyu Liu, Hao Li, Zhigang Zhao, Zhiyun Lu, Yan Huang, Dingguo Xu

**Affiliations:** ^1^MOE Key Laboratory of Green Chemistry and Technology, College of Chemistry, Sichuan University, Chengdu, China; ^2^College of Chemistry and Environment Protection Engineering, SouthWest University for Nationalities, Chengdu, China; ^3^Department of Chemistry, University of Science and Technology of China, Hefei, China

**Keywords:** organic solar cell, squaraine, DFT, D-A-D' framework, open-circuit voltage, short-circuit current density, fill factor

## Abstract

Squaraine core based small molecules in bulk heterojunction organic solar cells have received extensive attentions due to their distinguished photochemical properties in far red and infrared domain. In this paper, combining theoretical simulations and experimental syntheses and characterizations, three major factors (fill factor, short circuit and open-cirvuit voltage) have been carried out together to achieve improvement of power conversion efficiencies of solar cells. As model material systems with D-A-D' framework, two asymmetric squaraines (CNSQ and CCSQ-Tol) as donor materials in bulk heterojunction organic solar cell were synthesized and characterized. Intensive density functional theory computations were applied to identify some direct connections between three factors and corresponding molecular structural properties. It then helps us to predict one new molecule of CCSQ'-Ox that matches all the requirements to improve the power conversion efficiency.

## Introduction

Compared with polymers as donor materials in organic solar cells (OSCs), small organic molecules were developed even faster due to the several advantages, high purity, tunable electronic specialties and better device reproduction (Kan et al., 2014, 2015; Fan and Zhu, 2015; Collins et al., 2017). Moreover, solution-processed bulk heterojunction (BHJ) OSCs have great potential in realizing commercial solar cell devices due to the unique features like flexibility over a large area and low-cost fabrication (Huang et al., 2014). However, it is still challengeable to improve the power conversion efficiency (PCE) even higher to be commercialized although lots of efforts have been made to the synthesis (Li N. et al., 2013; Mulligan et al., 2014; Zhang et al., 2017).

Inspired by donor-acceptor (D-A) copolymers leading solar cells to 5~6% PCE (Peet et al., 2007; Park et al., 2009), where donor and acceptor unit are respectively conjugated electron-rich and electron- deficient moieties, the breakthroughs in PCE for small molecule donors were then achieved (Li et al., 2010). Moreover, such structural framework have been expanded to D-A-D, A-D-A, A-D-D-D-A, and A-π-D-π-A patterns, even their oligomers (Collins et al., 2017). It is notable that the asymmetric derivatives have a larger transition dipole moment so that they have higher performance than those centrosymmetric ones. Among those small molecules used in the BHJ OSCs, squaraine core based molecules have attracted extensive attentions due to their high extinction coefficients and relatively strong absorption in the far red and infrared domain (Chen et al., 2015; Gsänger et al., 2016), and thus have good overlap with solar spectra. In particular, squaraines have been demonstrated to have excellent thermal and (photo-) chemical stability as the dye and pigment chromophores. Squaraines can be generally assigned to a D_L_-A-D_R_ system. The center four-ring part behaves as an electron-acceptor, which is covalently linked to two electron donators. They can be accessible by using straightforward synthetic protocols. Potentials for commercial applications are thus highly expected. Further, asymmetrical squaraines (ASQs) bearing D-A-D' molecular frameworks can afford more room for molecular tailoring than symmetrical squaraines (SSQs) (Pandey et al., 2011). Our group reported an breakthrough in BHJ OSCs by using ASQs as electron donors (PCE_max_ = 6.0%) (Yang et al., 2015), although it is lower than other small-molecule including D-A structural frameworks based devices (10%) (Ni et al., 2015; Collins et al., 2017). In short, it is valuable to explore how the structural details of D-A-D' framework molecules as electron donor affect the performance in BHJ OSCs.

Albeit the PCEs of organic photovoltaic (OPV) cells have been improved to some extent, it is still far from commercial application in comparison with 22.1% PCE of lead halide perovkite based thin-film solar cells (Xiao and Yan, 2017). Further improvement in OPV performance is necessary. Basically, the PCE of a photovoltaic cell can be expressed as

(1) $$\begin{array}{r} PCE=\frac{{FF\cdot V}_{oc}{\cdot J}_{sc}}{I_{s}}, \end{array}$$

in which *J*_*SC*_ means the short-circuit current density, *V*_oc_ denotes the open-circuit photovoltage, *FF* is the fill factor, and *I*_S_ represents the intensity of incident (Bérubé et al., 2013). Clearly, to get a better PCE, none of these four components can be ignored in the material design.

Generally, the *V*_oc_ is related to the energy difference between the highest occupied molecular orbital (HOMO) of the donor material and the lowest unoccupied molecular orbital (LUMO) of the acceptor material. It is also affected by the rates of carrier generation and recombination, the presence of energetic tail states or trap states (Lange et al., 2013; Sweetnam et al., 2014). The *J*_*SC*_ is defined as the integration of the external quantum efficiency (EQE) along the wavelength across the solar spectrum (Bérubé et al., 2013). The EQE can be further divided into five fundamental processes,

(2) $$\begin{array}{r} \eta_{EQE}=\eta_{A}\eta_{ED}\eta_{CD}\eta_{CT}\eta_{CC} \end{array}$$

in which each term has the name of light absorption (η_A_), exciton migration (η_ED_), exciton dissociation or charge separation (η_CD_), charge transport (η_CT_), and charge collection to the electrodes (η_CC_) (Li, 2012). Therefore, to get better *J*_SC_, we should consider the combination of all five terms. Meanwhile, to achieve a high *FF* for BHJ OSCs, more efforts should be put on the device features, e.g., domain size or purity, gradated BHJ structures and π stacking distance or direction (Jao et al., 2016).

In contrast to experimental efforts in understanding fundamental mechanisms responsible for the photovoltaic properties of materials in OSCs, theoretical computational approaches represent an alternative way to establish the structure-property relationships. (Mennucci, 2015; Brückner and Engels, 2016; Volpi et al., 2016a,b; Alessandri et al., 2017; Brückner et al., 2017). Clearly, based on Equations (1, 2), various factors should work together to affect the PCE. However, rare work in material structural design includes all fundamental terms. Therefore, the objective in this work is to find the principle structural factors that regulate the fundamental photochemical properties. The asymmetrical squaraines (ASQs) with Fullerene based BHJ OSCs will be used as the example material systems.

**Figure 1:**
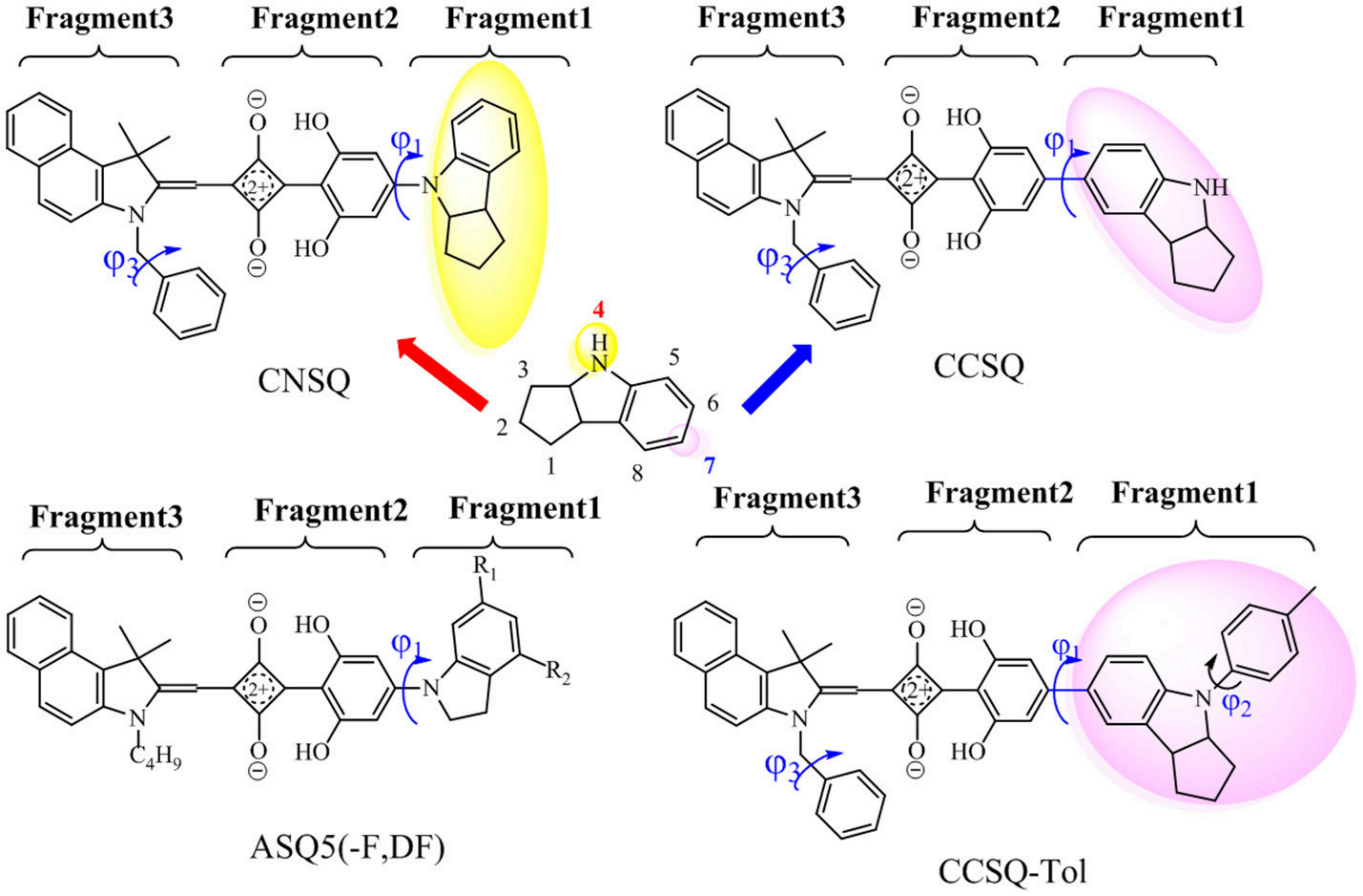
Investigated molecules [CNSQ, cCSQ, CCSQ-Tol and ASQ5(-F, DF); Yang et al., 2015] and definitions of three fragments used in this work.

In our previous work (Yang D. et al., 2014; Yang J. et al., 2014; Yang et al., 2015), a series of ASQs bearing benzindole-squarate-4-amino-2, 6-dihydroxyphenyl skeleton with different N-substitution like carbazole, indole, and indoline, were synthesized and characterized. All of them have good solubility, high film quality and achieved PCEs in range of 1.54–4.29% for solution-processable ASQ-based BHJ-OSC. Specifically, indoline group seems to be one of good candidates for further modification to get better solar cell performance. As shown in Figure 1, indoline subunit has two different linking positions (4 or 7), which can be attached by different groups. Particularly, the 4-position can be linked to a squaraine 4-member ring via dihydroxyphenyl group, which is denoted as C-N linkage. The 7-position can be linked to an electron-deficient core, namely C-C linkage. Indeed, thanks to different electronic structures and molecular skeleton of groups linked at 4 or 7 position, distinguished device performances in these two kinds of materials based dye-sensitized solar cell (DSSC) can be found like absorption, aggregation, or morphology (Li G. et al., 2013; Yang D. et al., 2014).

In this work, two asymmetrical squaraines bearing indoline moiety were designed with different linkages, in which the 4- and 7-positions of indoline were linked to squarate core via dihydroxyphenyl group. They are defined as CNSQ and CCSQ, respectively. An additional molecule, namely CCSQ-Tol, was also synthesized by us with the H atom of CCSQ replaced by a toluene group as shown in Figure 1. In addition, three other well-characterized ASQ derivatives have been reported by us (Yang et al., 2015). Their good photophysical performances have been considered as the result of particular π-π stacking effects. Therefore, with all these different squaraine based donor molecules systematically theoretical computations can help us establish reliable connections between structural elements and the fundamental factors of PCE in BHJ OSCs. This can provide more opportunities to enhance photophysical properties of OSCs based on asymmetrical squaraines core.

## Materials and methods

First of all, only two target molecules CNSQ and CCSQ-Tol, due to instability of CCSQ's N-H bond in BHJ OSC, were synthesized and characterized. All experimental details can be found in Supplementary Material. As we can see in Table 1, the PCEs for two donor molecules, CNSQ and CCSQ-Tol, were not significantly improved as expected. In addition, the device based on CCSQ-Tol has higher *J*_SC_ than that based on CNSQ. Of course, some apparently linking topological or N-H substitution effects can be identified. Theoretically, the molecule of CCSQ is also included in our simulation. In addition, in order to shed more lights to this interesting issue, more molecules based on asymmetric squaraines core, e.g., ASQ5, ASQ5-F, and ASQ5-DF, have also been evaluated in this work.

**Table 1:** Optimized OSC performance data with blended ratio of ASQs:PC_71_BM = 1:5 (w/w).

**Active layer (w/w)**	***J_*sc*_***	***V_*oc*_***	**FF**	**PCE (%)**
	**(mA cm^−2^)**	**(V)**		
CNSQ:PC_71_BM = 1:5	9.93	0.84	0.47	3.91 (3.76)*^*a*^*
CCSQ-Tol:PC_71_BM = 1:5	10.50	0.80	0.41	3.41 (3.24)*^*a*^*
ASQ5:PC_71_BM = 1:5	10.98	0.82	0.45	3.76(3.64)*^*b*^*
ASQ5-F:PC_71_BM = 1:5	11.18	0.88	0.46	4.26(4.18)*^*b*^*
ASQ-DF:PC_71_BM = 1:5	11.56	0.93	0.46	4.71(4.60)*^*b*^*

a *Report as the form of best value (average value), average value obtained from at least 16 devices*.

b *Reported data are extracted from Yang D. et al. (2014) with the first data are the best PCEs obtained and numbers in parentheses are average values over 12 individual devices*.

Previous studies (Yang D. et al., 2014; Yang J. et al., 2014; Yang L. et al., 2014) suggested that the hydroxyl groups of phenyl rings on the asymmetric squaraine system can form intramolecular hydrogen bonds with oxygen atoms on squarate core. A co-planar topology is displayed in Figure S9. Therefore, all targeted asymmetrical squaraines can be divided into three fragments with D-A-D' framework according to Figure 1. Specifically, **Fragment 1** as the donor unit contains the indoline moiety with different linking topology or substitution; **Fragment 2** as the accept unit includes square acid and dihydroxyl benzene; and **Fragment 3** as the donor unit denotes the benzoindole derivative. Since the linking topological and substitution effects mainly occur in **Fragment 1** of D-A-D' system, the torsion angle between **Fragment 1** and **Fragment 2** denoted as ϕ_1_ and the covalent bond distance between them denoted B_12_ need more attentions. Two additional torsion angles of ϕ_2_ and ϕ_3_ have also been defined as shown in Figure 1 for further discussion.

To fully understand the relationship between the molecular structures and corresponding photophysical properties of target materials, intensive density functional theory (DFT) calculations were then applied. The Becke3-Lee-Yang-Parr (B3LYP) exchange-correlation functional and a standard basis set of 6-31G(d) were first used in the full geometry optimization to locate the ground state (S_0_) configurations. Subsequently, the geometric optimization and property analyses of electronic excited states of S_1_ for all molecules were carried out at B3LYP/6-31G(d) and M06-2X/6-31++G(d, p) levels of theory within TD-DFT (Scalmani et al., 2006) framework. Meanwhile, to account for the solvent effects, the polarizable continuum model (PCM) (Tomasi et al., 2005) was applied using chloroform as the solvent. All DFT computations were performed using the Gaussian 09 suite of program (Frisch et al., 2009). The relationship between the structures and properties were based on electronic structure analyses, which were achieved using the package of Multiwfn (Lu and Chen, 2012). In addition, the ESP plots were rendered by Visual Molecular Dynamics (VMD) software (Humphrey et al., 1996).

## Results and discussion

### Geometry and spectra

For the systems investigated in this work, the method of B3LYP/6-31G(d) has good performance for the ground state of all target molecules, but it failed to correctly characterize the electronic excitation of the asymmetric squares. According to Table 2, the calculated stokes shifts of CNSQ and CCSQ-Tol at M06-2X/6-31++G(d, p) level are 0.09 and 0.20 eV, which is consistent with experimental 0.06 and 0.14 eV despite a little bit red-shift. However, such 2 values at B3LYP/6-31G(d) are 0.21 and 0.22 eV, which is too close and inconsistent with experimental observations. Basically, larger stokes shifts usually occurs on account of more conformational differences between ground and excited states (van Duren et al., 2004). According to Table S1, we can find that CCSQ-Tol exhibits much larger conformational change than CNSQ from states of S_0_ to S_1_, e.g., the torsion angle ϕ_1_ (8° vs. 2.7°) and the bond distance B_12_ (0.021 vs. 0.0 Å). Clearly, results based on B3LYP/6-31G(d) cannot reproduce this geometrical variation. Therefore, M06-2X/6-31++G(d,p) with solvent effect was then selected for all density functional theory calculations if not otherwise stated.

**Table 2:** The calculated absorption and emission spectra at B3LYP/6-31G(d) and M06-2X/6-31++G(d, p) level in chloroform in comparision with experimental data^*^.

		**CNSQ**		**CCSQ-Tol**		**CCSQ**	
		**S_0_->S_1_**	**S_1_->S_0_**	**S_0_->S_1_**	**S_1_->S_0_**	**S_0_->S_1_**	**S_1_->S_0_**
B3LYP/6-31G(d)	λ^cal^(eV)	2.05	1.84	1.79	1.57	1.94	1.65
	*f*	1.994	1.943	1.486	1.160	1.683	1.325
M06-2X/6-31++G(d,p)	λ^cal^(eV)	2.04	1.95	2.08	1.88	2.11	1.90
	*f*	2.148	2.138	2.099	2.322	1.958	2.168
Experiment	λ^exp^(eV)	1.81	1.75	1.78	1.64	1.81	1.75
	log ε	5.36		5.10			

* *f, the oscillator strength; ε, molar extinction coefficient*.

As we have mentioned above that there two linking sites on indoline group, we noticed that this could cause large geometric variations for both S_0_ and S_1_ states for all three molecules of CCSQ, CCSQ-Tol, and CNSQ. According to Table S1, we can also find that the substitution of tolune group to CCSQ cannot change the overall geometry too much. Moreover, these different geometries cause distinct features in crystal structures. As shown in Figure S1, CNSQ shows stronger aggregation with π-π distance of 3.93 Å (2θ = 21.88°), while CCSQ-Tol exhibits weaker reflection peaks at 2θ = 23.78°, corresponding to π-π or π-edge distance of 3.68 Å. Unfortunately, efforts to cultivate single crystal for CCSQ-Tol finally failed. The experimentally monomer and superposition structures have been shown in Figure S2. In particular, the calculated torsion angles ϕ_1_ in chloroform for CNSQ is 13.1°, which is close to 4° in its X-ray structure. The nearly planar conformation of CNSQ in crystal structure might be due to the π-π stacking effects. Compared to CNSQ, much poorer π-π stacking effect for CCSQ-Tol can be seen based on the geometrical optimization. Indeed, ϕ_1_ for CCSQ-Tol is calculated to be 31.6°, and ϕ_2_ of 38.9°Can be also obtained.

Such poor π-π stacking effect can be further confirmed by the experimental absorption wavelength in chloroform and film (see in Table S4). In chloroform solution, the maximum absorption peaks of CNSQ and CCSQ-Tol are around 690 nm. The CNSQ shows higher molar extinction coefficient (log ε = 5.36) when compared to CCSQ-Tol (log ε = 5.10). However, CNSQ shows a typical sharp absorption band, which is much narrower than that of CCSQ-Tol, evidenced by full width at half maximum (FWHM) of 38 nm (CNSQ) vs. 108 nm (CCSQ-Tol). Relative to their absorption spectra in solution, CNSQ exhibits a more pronounced red-shift (43 nm) and broad absorption spectrum in thin film than that of CCSQ-Tol, which can be attributed to strong aggregation in solid state. Interestingly, the absorption spectrum of CCSQ-Tol in thin film is only slightly red-shift (16 nm), which indicates the weaker packing (Pommerehne et al., 1995; Wei et al., 2011). Nevertheless, the absorption spectrum of CCSQ-Tol still cover a wide range of 430–860 nm with a relatively broad FWHM of 195 nm, which is desirable for efficient light harvest.

### Short-circuit current density

Significantly different structural and π-π stacking features caused by linking topological and substitution effect of CNSQ, CCSQ and CCSQ-Tol can be observed based on experimental and theoretical characterizations. Therefore, it would be important to explore how they affect the fundamental factors of PCE, *esp*., for short-circuit current density (J_SC_). Basically, *J*_SC_ is affected by five factors as shown in Equation (2). The property of η_CC_ to the electrodes is considered to be only related to the device fabrication, but not with the molecule itself. Thus, it was simply ignored here. We will then discuss other four terms individually below, and try to find out which term has dominant contribution to *J*_SC_.

#### Electronic transition analysis

The electronic transition properties for CNSQ and CCSQ-Tol are presented in Table 3. CNSQ has the largest transition dipole moment μ_tr_ (6.34 a.u.) and the smallest distance index Δr (1.90 Å) compared to CCSQ and CCSQ-Tol. Generally, norm of transition dipole moment μ_tr_ is related with the oscillator strength, which can explain the strength of light absorption (η_A_). The distance index, Δr, between centroids of hole and electron usually represents a measure of intra-molecular charge-transfer (iCT). The smaller Δr is, the more likely the excitation is a local excitation mode (LE). To this point, the excitation for CCSQ-Tol is closer to an iCT mode. The CNSQ with more LE transition mode should have larger light absorption η_A_. This is consistent with the fact that CNSQ in chloroform solution has higher molar extinction coefficient (log ε = 5.36) when compared to CCSQ-Tol (log ε = 5.10). All these data were summarized in Figure S4 and Table S4. In last part, we have already suggested the substitution has little effects on the overall geometry of molecular moieties. Furthermore, peak positions of adsorption and emission spectra are close for CCSQ and CCSQ-Tol, shown in Figure S4.

**Table 3:** Electron transition properties.

	**μ_tr_(a.u.)**	**Δμ(a.u.)**	**RMSD of electron (Å)**	**RMSD of hole (Å)**	**Δr(Å)**
CNSQ	6.34	3.40	3.97	5.05	1.90
CCSQ-Tol	6.15	3.48	3.96	5.37	6.35
CCSQ	6.05	2.37	3.90	4.96	4.36

*μ_tr_, norm of transition dipole moment*.

*Δr, distance between centroid of hole and electron*.

*Δμ, variation of dipole moment with respect to ground state*.

**Figure 2:**
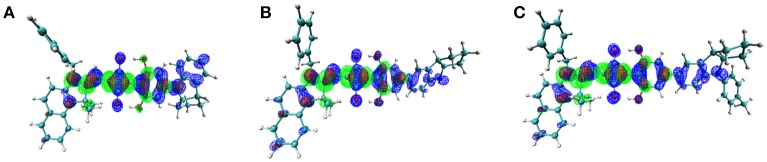
Electron (green), Hole (blue), and Overlap (red) Distribution for **(A)** CNSQ, **(B)** CCSQ, and **(C)** CCSQ-Tol.

On the other hand, to characterize the carrier transport property η_CT_, the hole mobility of the pristine and blend films (ASQs: PC_71_BM = 1: 5) were measured by the space charge limited current (SCLC) method, and the structures were ITO/MoO_3_ (8 nm)/active layer (80 nm)/Au (100 nm) (see Figure S6 in Supplementary Material). In pristine film, the hole mobility of the CCSQ-Tol (2.21 × 10^−5^ cm^2^ V^−1^ s^−1^) is slightly higher than that of CNSQ (1.68 × 10^−5^ cm^2^ V^−1^ s^−1^), which is consistent with the RMSD values of hole distribution according to Table 3. RMSD of hole or electron distribution denotes the region that an electron leaves or enters in the single-electron excitation process between S_0_ and S_1_. To get a better view of the electronic structures, we then plotted the hole, electron distributions and their corresponding overlaps for all three molecules in Figure 2. Obvious difference for the hole distributions (blue marked region) occurs in **Fragment 3**. From Table 3, CCSQ-Tol has broader hole distribution than CNSQ, while all three molecules have similar electron distributions. It could thus be expected that the development of a larger hole distribution for donor-materials could lead better η_CT._ However, in blend film, the hole mobility of the CCSQ-Tol: PC_71_BM = 1:5 film (1.46 × 10^−6^ cm^2^ V^−1^ s^−1^) is much lower than that of CNSQ: PC_71_BM film (3.72 × 10^−6^ cm^2^ V^−1^ s^−1^).

Obviously, although larger values of η_A_ and η_CT_ for CNSQ than CCSQ-Tol have been identified via experimental measurements and theoretical computations, the *J*_SC_ has a reverse performance between two molecules. This could tell us that we could not expect to enhance *J*_SC_ (Table 1) via increasing η_A_ and η_CT_. In other words, simple adjustment of the molecular topology might not work here, esp., for asymmetric squaraine based OSCs. We might focus on other two parameters of η_ED_ and η_CD_.

#### Electrostatic analysis

There are no effective experimental methods that can be used to measure the η_*CD*_ and η_ED_. Theoretical simulation represents an alternative way to address this issue. Basically, there are two kinds of excitons in OSCs, molecular exciton and charge-transfer exciton (Zhugayevych and Tretiak, 2015; Vandewal, 2016). The exciton migration of η_ED_ is related to the former case, while the exciton dissociation of η_*CD*_ is related to the latter case. According to the electrostatic interaction, monomers at S_0_ or S_1_ state tend to contact each other in complementary manner of electrostatic potential (ESP), i.e., the electron deficient part prefers interacting with the electron rich part. Therefore, the ESP information of both ground and excited states could give a hand to connect two factors of *J*_SC_ (η_ED_ and η_CD_) with structural features of asymmetric squaraines.

**Figure 3:**
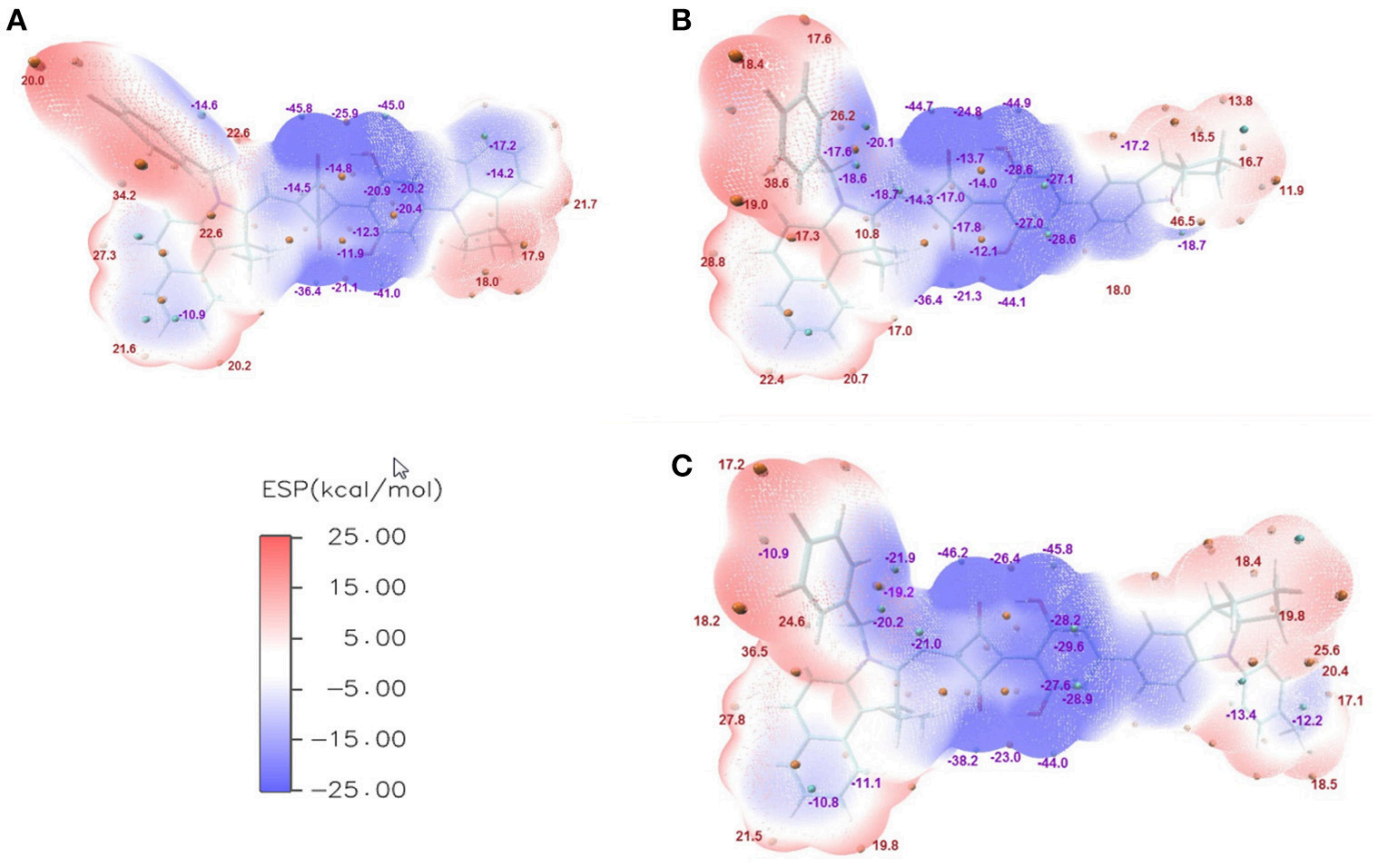
ESPs on the vdW surface for **(A)** CNSQ, **(B)** CCSQ, and **(C)** CCSQ-Tol at vertical excited states with blue denoting extremely electron-deficient region (corresponding to negative value), red denoting electron-rich region (corresponding to positive value) and white denoting neutral region. The cyan and orange spheres correspond to ESP surface minima and maxima, respectively. For simplification, only the most negative and positive values have been labeled.

The ESPs of CNSQ, CCSQ, and CCSQ-Tol were plotted for vertical excited states in Figure 3 at the 0.001 electron/bohr^3^ isosurfaces of electron density. The color ranges from −25 to 25 kcal/mol. From Figure 3, the ESP values are negative inside the aromatic group or on electron-withdrawn group, featuring electron-deficient region. ESP values are positive on hydrogen atoms of aromatic group or atoms on alkyl group, which means an electron-rich feature. The electro-deficient region covers not only the square acid but also the dihydroxyl benzene group in the asymmetric squaraine. The global positive maxima for three molecules can be located nearly at the benzoindole derivative, while much smaller local maxima with positive ESP values can be found at the right counterpart. It should be noted that the excited state discussed here is at Franck-Condon region via vertical excitation since the molecule of BHJ OSC in condensed phase might not have sufficient time to change its conformation after light absorption.

For η_ED_, the exciton could transfer (or hop) from one molecule to the other until it arrives to the donor/acceptor (D/A) interface. The interaction type between two molecules is the key during this process. Generally, it tends to be achieved in the direction of electron transferring from the most electron-rich region of the excited state to the most electron deficient region of the ground state. Or in the reverse direction of hole transferring, it is from the most electron deficient region of the excited state to the most electron-rich region of the ground state. Hence, the electron transferring ability (denoted as *T*_*e*_) and the hole transferring ability (denoted as *T*_*h*_) can be used to elucidate exiton transfer (η_ED_). According to the calculation, CCSQ-Tol has larger *T*_*e*_ and *T*_*h*_ (83.2 and 90.3 kcal/mol) than CNSQ (79.8 and 83.2 kcal/mol), which is consistent with the values of observed *J*_*SC*_. That means the property of η_ED_ shows similar trend with *J*_*SC*_ for the squaraine based system_._

On the other hand, formation of charge-transfer (CT) excitons also depends on electrostatic interaction between the most electron-rich region of donor molecules and the most electron deficient region of acceptor molecules at both excited state. The value of η_*CD*_ is mainly affected by formation of CT excitons. Therefore, a more favorable η_*CD*_ should be connected with the larger ESP extrema of electron-rich region on donor molecule. Here, the most positive extrema of the electron-rich region is defined as *Xe*. In donor materials investigated in this work, CCSQ-Tol has a larger *Xe* (36.6 kcal/mol) on electron rich region than CNSQ (34.2 kcal/mol). That might partially mean η_CD_ also has the same trend with *J*_SC_, although current samples are not sufficient.

According to above analyses, compared to η_A_ and η_CT_, the improvement of both η_*ED*_ and η_*CD*_ seems to be feasible to enhance *J*_SC_ for the squaraine based materials. Now the question is which one is dominant. To address this issue, we then include three other ASQ based molecules (ASQ5, ASQ5-F, ASQ5-DF) in our computations, which have been synthesized and characterized by us (Yang et al., 2015). They all show some good performances of OSC devices. Since the ASQ5 holds nearly the same topology as CNSQ/CCSQ, it is then expected to provide us more information about how morphology or electronic structures affecting material properties.

**Figure 4:**
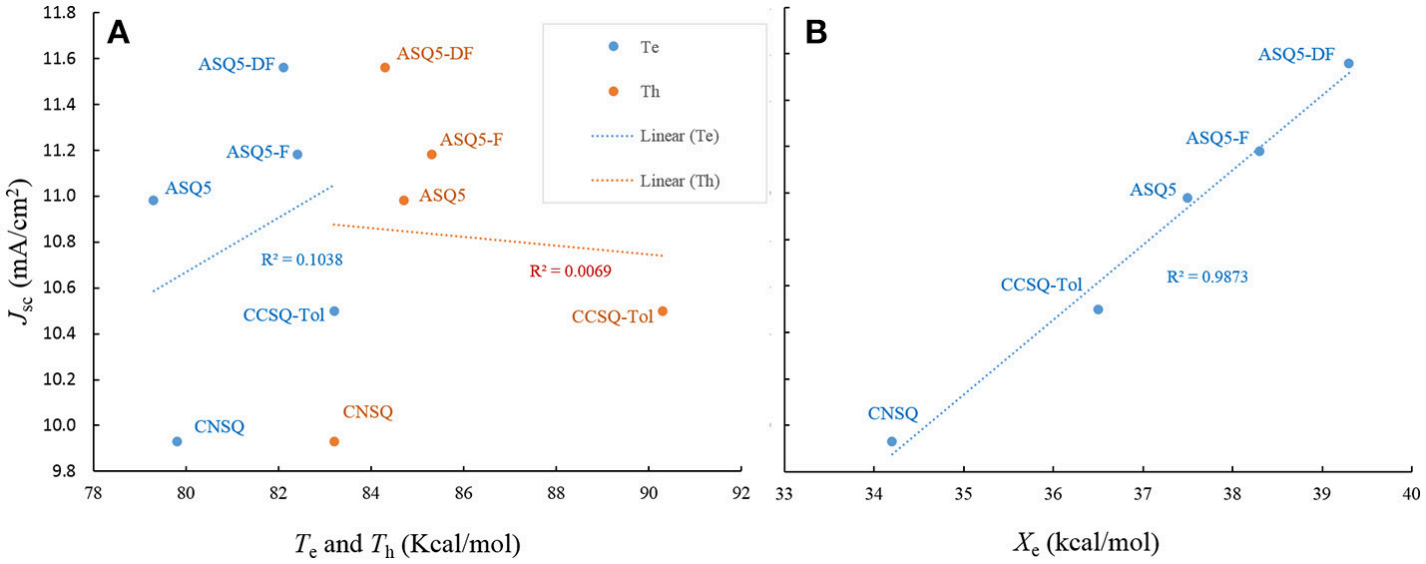
The *J*_sc_ as a function of electron/hole transferring ability (*T*_e_/*T*_h_) **(A)**, and electron-rich extreme value on ESP surface *X*_e_ **(B)**.

Like CNSQ and CCSQ-Tol, η_A_ and η_CT_ of the devices based on these three donor molecules ASQ5, ASQ5-F, and ASQ5-DF were also shown to show opposite performance with *J*_SC_. Therefore, we can rule out the efforts to improve *J*_SC_ with helping of either η_A_ or η_CT_. As we have mentioned above, the variation tendency of *T*_e_ and *T*_h_ matches the trend of *J*_SC_ of CCSQ-Tol and CNSQ. However, no such principle can be found for the fluorine substitution cases. According to Table S6, we could see that there is no connection between *T*_e_ or *T*_h_ with *J*_SC_. We then plot the possible correlation in Figure 4A. Clearly, we could not see any real correlation between *T*_e_ (or *T*_h_) and *J*_SC_ for all five systems investigated here. In other words, we also could not expect to improve *J*_SC_ by regulating exiton transfer or η_ED_. Quite interestingly, similar with CNSQ and CCSQ-Tol, values of η_*CD*_ of ASQ5, ASQ5-F, and ASQ5-DF have the same trends with *J*_SC_ (see more details in Table S6). In particular, with the fluorine substitution, *J*_SC_ improves as well. Accordingly, the *X*_*e*_ also increases, i.e., 37.5 kcal/mol for ASQ5 and 38.3 kcal/mol for ASQ5-F, and 39.3 kcal/mol for ASQ5-DF. Moreover, a perfect linear correlation between *J*_SC_ and *X*_e_ can be established according to Figure 4B. Since the *X*_e_ value is directly connected with the η_CD_, we could safely conclude that a better η_CD_ can lead a higher *J*_SC_, *esp*., for asymmetric squaraine based OSC devices.

### Analysis of V_OC_

It has been well accepted that a higher *V*_OC_ in OSC usually correlates with higher HOMO level for donor material molecules or lower LUMO level for acceptor material molecules. Considering the same acceptor material molecule used in this work, it is not necessary to pay much attention to LUMO. First of all, the HOMO levels of CNSQ and CCSQ-Tol are calculated to be −6.14 and −6.12 eV, respectively. The calculated absolute values are larger than experimental data (see details in Table S4). From Table 1, we still can find their difference is consistent with *V*_OC_, i.e., a bit higher *V*_OC_ (0.84 V) for the CNSQ-based OPV device than CCSQ-Tol (0.80 V) based one. On the other hand, considering much lower HOMO level of −6.30 eV for CCSQ and a little bit smaller value of *V*_OC_ for CCSQ-Tol based device, it can be postulated that the backbone framework of CCSQ has positive contributions to *V*_OC_ compared with CNSQ, while the toluene group contributes negatively to *V*_OC_.

**Figure 5:**
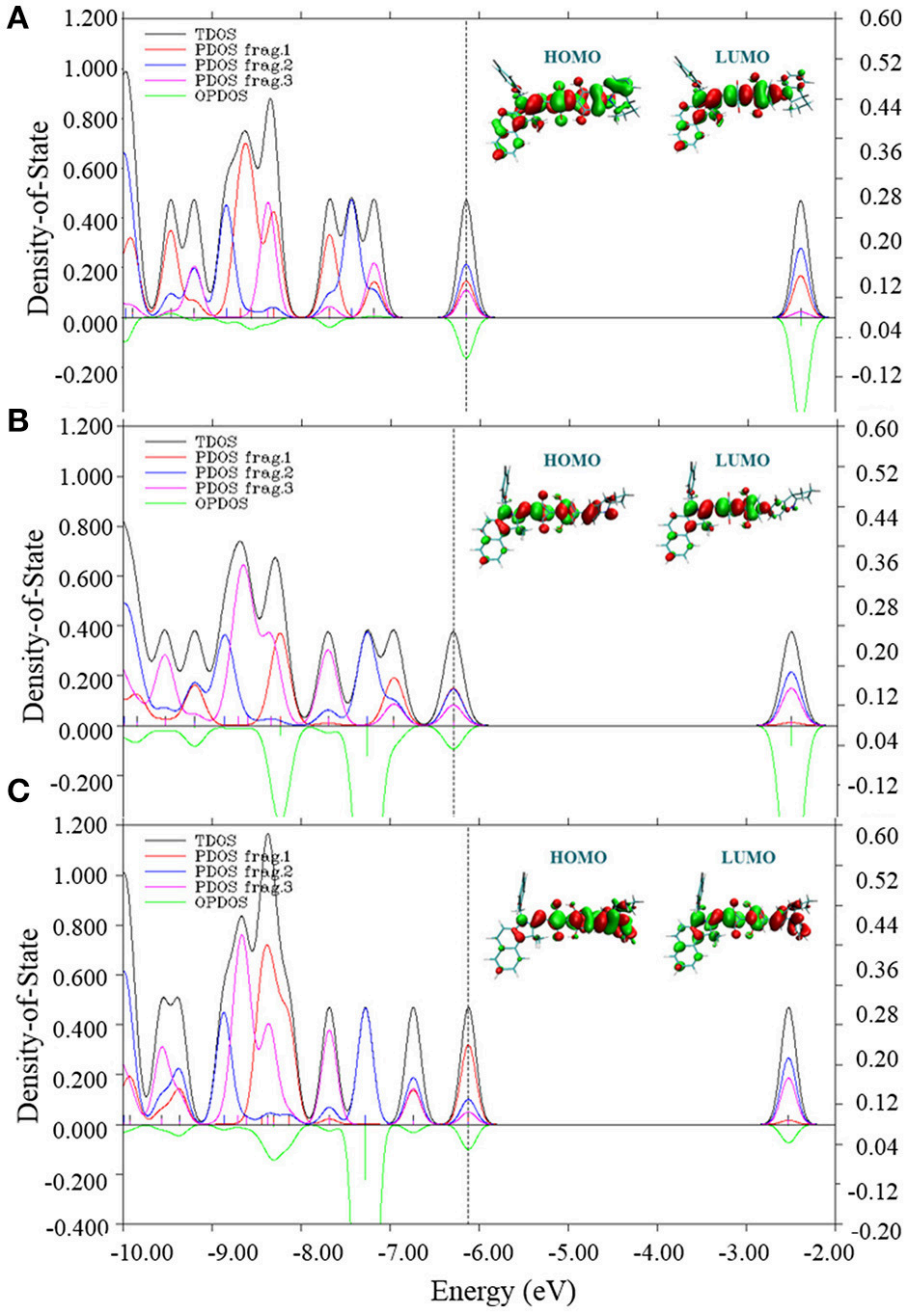
Total density of states (TDOS), partial DOS (PDOS) and overlap DOS (OPDOS) between fragment 1 and fragment 2 with HOMO and LUMO for **(A)** CNSQ, **(B)** CCSQ, and **(C)** CCSQ-Tol.

To further understand the effects of electronic structures of all three molecules, total (TDOS), partial (PDOS) and overlap density of states between **Fragment 1** and **Fragment 2** (OPDOS) were depicted in Figure 5. In fact, suitable electronic structure of material is crucial to determine fundamental photophysical features of OSCs. The PDOS distribution clearly suggests that three fragments of CNSQ and CCSQ contribute similarly to their HOMOs of TDOS. However, **Fragment 1** of CCSQ-Tol contributes much more to HOMO than **Fragments 2** and **3**. It further indicates that the toluene group has negative contribution to *V*_oc_. On the other hand, the overlap DOS between **Fragments 1** and **2** show negative contributions to the frontier orbitals, which clearly indicates that those molecular orbitals are antibonding orbitals. To this point, we might say that the N-substitution on the position of the **Fragment 1** of CCSQ could significantly affect the *V*_OC_.

### Analysis of fill factor

**Figure 6:**
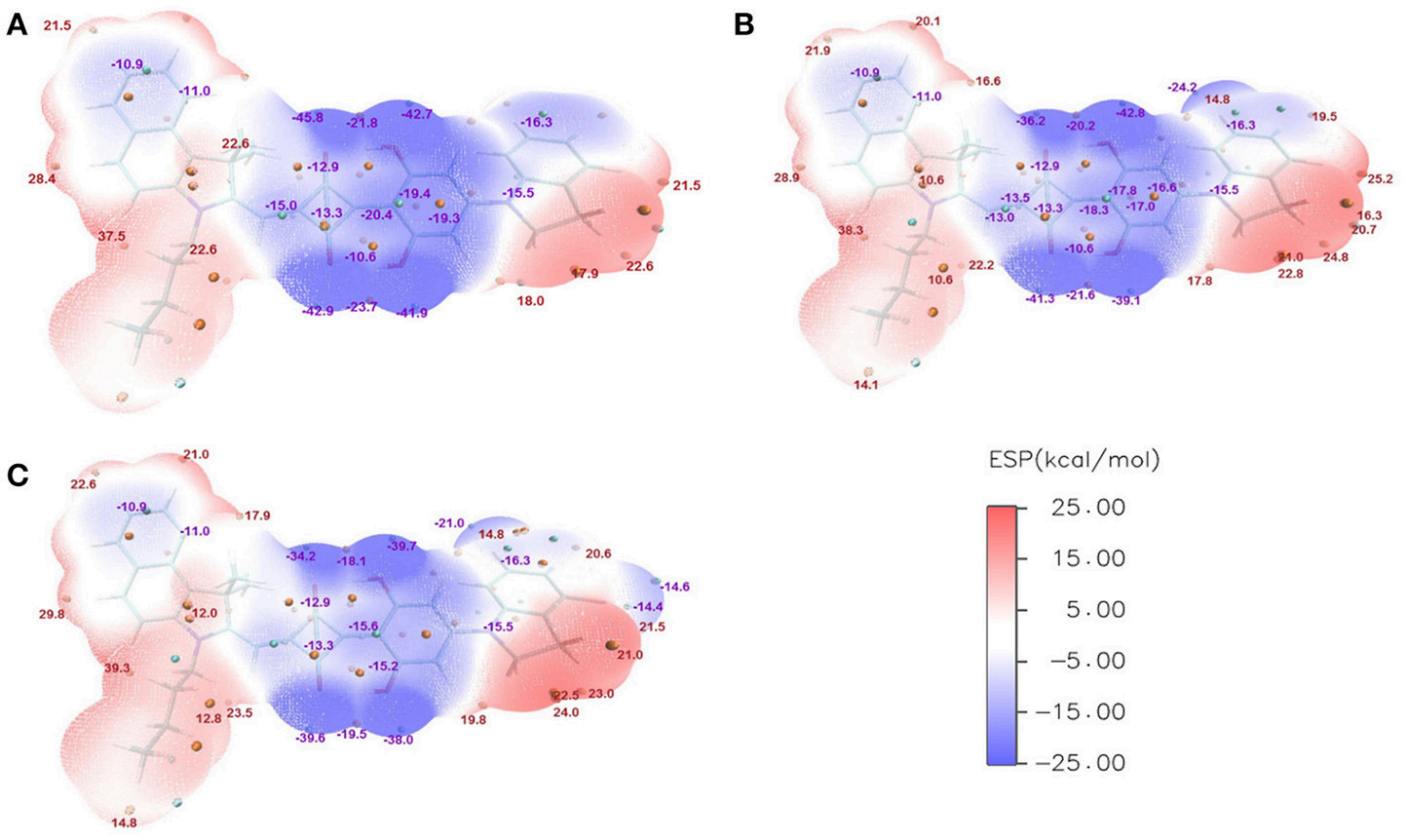
ESPs for **(A)** ASQ, **(B)** ASQ-F and **(C)** ASQ-DF at vertical excited state.

Gupta et al. (2010) have suggested that the *FF* depends on combination of some intrinsic device properties including the product of mobility and lifetime of the bulk material, thickness of the active-polymer layer and morphology of the cathode/polymer interface. Since all device parameters based on these donor materials are measured under the same conditions, the *FF* mainly relies on the charge carrier mobility of the electron (acceptor) and hole (donor) transport materials. It also improves with the presence of crystalline or relatively pure aggregate domains in the BHJ blend. In the system of ASQ5, we found that the *FF* value systematically increases with the fluorine substitution. Specifically, it is 0.51 for ASQ5, 0.52 for ASQ5-F, and 0.56 for ASQ5-D (Feng et al., 2015). These phenomena can be attributed to their special packing patterns between layers in crystals, e.g., **Fragment 3** of ASQ5 interacts with **Fragment 2** of the nearest neighbor layer, whereas **Fragment 3** of ASQ5-DF has contacts with **Fragment 1** of the nearest neighbor layer. To understand this issue, we then plotted their ESPs in Figure 6. The **Fragment 1** is generally electron rich, except the position at the fluorine substitution, which appears to be electron deficient. It has been suggested by us (Yang et al., 2015) that this special electronic distribution should be the reason to cause different crystal packing patterns between ASQ5 and ASQ5-DF. To find out the direct relationship between molecular structure and the *FF* value, we summarized selected geometric parameters for three ASQ5 based molecules in Table 4. The biggest variation can be found is in ϕ_1_, which increases from 25.2 to 28.9° with fluorine substitution. Thus it could be deduced that the larger ϕ_1_ will benefit the complementary approaching between **Fragments 1** and **3** of different molecules due to the substitution on N atom of **Fragment 3**. Meanwhile, if we recall the torsion angles of ϕ_1_ for CNSQ and CCSQ-Tol in Table 4, we can easily find that the latter molecule has a larger ϕ_1_ but smaller *FF* value. The reason is that much less contact between **Fragment 3** and **Fragment 2** of different molecules can take place after toluene substitution on CCSQ. Clearly, our results can strongly support that the morphology of material plays a key role in regulating *FF* value.

**Table 4:** Theoretical characterizations based on molecular design (the data about all the structures has been listed here to be compared)^*^.

	**ϕ_1_(^°^)**	**HOMO(eV)**	**λ^abs^(eV)**	***f***	***X*_e_**
CNSQ	13.1	−6.14	2.04	2.148	34.2
CCSQ-Tol	31.6	−6.12	2.08	2.099	36.5
CCSQ	32.9	−6.30	2.11	1.958	37.6
ASQ5	25.2	−6.13	2.06	2.040	37.5
ASQ5-F	27.7	−6.20	2.08	1.999	38.3
ASQ5-DF	28.9	−6.28	2.11	1.959	39.3
CCSQ'-Ox	34.8	−6.47	2.21	1.854	40.2

* *ϕ_1_ is defined as the torsion angle between Fragment 1 and Fragment 2; f, the oscillator strength; λ^abs^, wavelength of absorption*.

Finally, we can summarize a basic principle to design a new donor molecule with D-A-D' electronic structure used in OSC devices here. The preferred molecules should have good intermolecular packing, bigger positive extremes on ESP at vertical excited state and lower HOMO energy level. With these basic rules, new molecules with better performances could be designed. Experimentally, CCSQ-Tol has better *J*_*SC*_ but poorer *V*_OC_ than CNSQ, which is consistent with our theoretical investigations presented above. However, the topological and substitution analyses do show that CCSQ has lower energy level of HOMO than CNSQ. We can simply deduce that the linking topology like CCSQ is beneficial to improve *V*_OC_, but the toluene substitution acts on the contrary.

**Figure 7:**
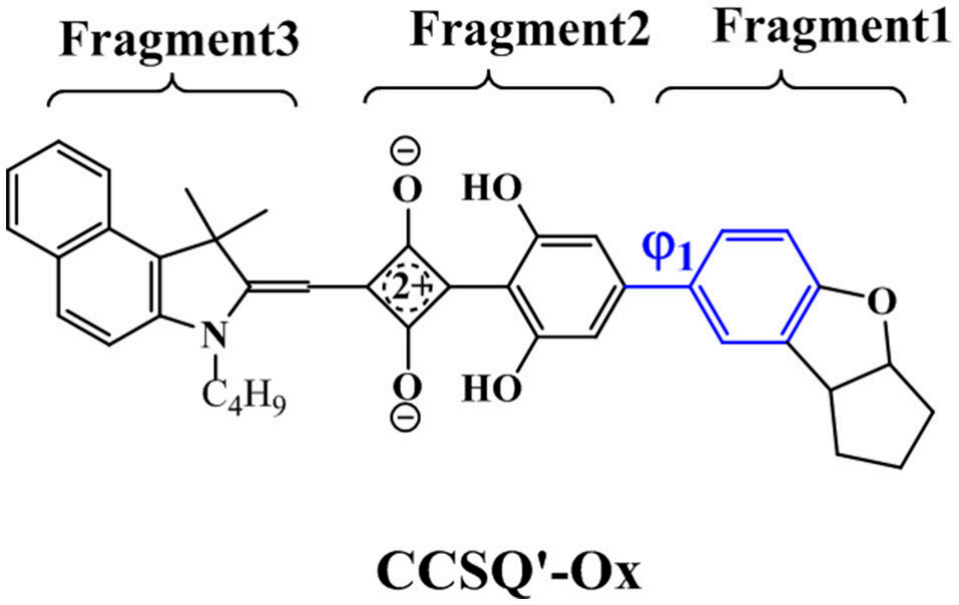
Newly designed molecule.

Further, the design of new material molecule should consider how to increase *FF*, which can be inspired by the case of ASQ5-DF. The fluorine substitution on the electron-rich region of asymmetric squaraine makes the atom at the linking position more electron deficiently, thus a better packing style between **Fragment 1** and **Fragment 3** can thereby be achieved. To match the packing pattern like ASQ5-DF, the newly designed CCSQ based molecule was developed with O group replaced by N group on **Fragment 1** on account of its strong electron-deficient property. In addition, the group of -CH_2_Ph was replaced with the group of -C_4_H_9_ on **Fragment 3**. This newly constructed molecule, namely CCSQ'-Ox, is depicted in Figure 7. According to Table 4, relatively large ϕ_1_ of 34.8°For CCSQ'-Ox can be found, which has been considered to be a sign for the good intermolecular stacking between Fragments 1 and 3. Meanwhile, the lowest HOMO at −6.47 eV for CCSQ'-Ox also means the best *V*_OC_ among molecules we have consulted in this work. Moreover, the biggest positive extrema of 40.2 kcal/mol on ESP for CCSQ'-Ox can be seen from Table 4. It is thus expected a larger *J*_SC_. Theoretically, the CCSQ'-Ox seems to include all three aspects that affect the final PCE of BHJ OSC, and thus represents one possible candidate to develop a new BHJ OSC with better properties. Experimental characterizations currently are underway in our lab.

## Conclusions

It has been well accepted that experimental and theoretical groups can work together to enhance the R&D speed of new materials. Our objective is to establish the reliable connection between intrinsic molecular structure and corresponding device performances of OSCs. For the donor molecules with D-A-D' framework, we have carried out systematically experimental and theoretical characterizations for a series of asymmetric squaraines.

Based on the previous work, we have designed three new molecules, namely CNSQ, CCSQ and CCSQ-Tol. Considering the stability of materials and devices, only features for CNSQ and CCSQ-Tol based devices were measured and characterized. The device based on CNSQ has higher PCE than that based on CCSQ-Tol. Combined experimental characterizations and theoretical calculations suggest that the improvement of the device performance cannot just depend on one single factor. Specifically, we can confirm that simple adjustment of the overall topology for the donor molecules might not work for improving *J*_SC_, we have to focus on regulating their intrinsic electronic structure properties. A linear correlation between *J*_SC_ and the positive extrema (*X*e) on ESP can be established. In summary, our simulations indicate that a good intermolecular stacking interaction is required for *FF*, the bigger positive extrema on ESP at vertical excited state is desired for improving *J*_SC_, and lower HOMO energy level is for better *V*_OC_.

Finally, based on our first principle computations, one new molecule, namely CCSQ'-Ox, was predicted with better values of *FF, V*_OC_, and *J*_SC_, which might have promising PCE. Of course, extensive experimental investigations should be required to confirm our prediction. It is also our hope to encourage improving device performance for new BHJ OSC with the help of theoretical simulations.

## Author contributions

JC, YH, and DX contributed conception and design of the study. QL, HL, ZZ, and ZL organized the database. JC performed the statistical analysis. JC wrote the first draft of the manuscript. JC, QL, YH, and DX wrote sections of the manuscript. All authors contributed to manuscript revision, read and approved the submitted version.

### Conflict of interest statement

The authors declare that the research was conducted in the absence of any commercial or financial relationships that could be construed as a potential conflict of interest.
