# Supplementary material for: Density Functional Theory Investigations of D-A-D' Structural Molecules as Donor Materials in Organic Solar Cell
Source: Front Chem. 2018 Jun 4;6:200. doi: 10.3389/fchem.2018.00200 (PMC5994543; doi:10.3389/fchem.2018.00200)
Supplement: Supplementary file 1 [file Presentation_1.pdf]

Supplementary Material

In Silico and Experimental Investigations of D-A-D´ Structural Molecules as Donor Materials in Organic Solar Cell

Junxian Chen[1,2], Qingyu Liu[1], Hao Li[3], Zhigang Zhao[2], Zhiyun Lu[1], Yan Huang[1*], and Dingguo Xu[1*]

[1]MOE Key Laboratory of Green Chemistry and Technology, College of Chemistry, Sichuan University, Chengdu, Sichuan, 610064, PR China

[2]College of Chemistry and Environment Protection Engineering, SouthWest Minzu University, Chengdu, Sichuan, 610061, PR China

[3]Department of Chemistry, University of Science and Technology of China, Anhui, Hefei 230026, P. R. China

*** Correspondence:**
Dingguo Xu, dgxu@scu.edu.cn
Yan Huang, huangyan@scu.edu.cn

1 **Experimental Information** 3

1.1 **Synthesis and Device fabrication** 3

1.2 **Materials** 4

1.3 **Instruments and measurements** 8

2 **Supplementary Figures and Tables** 10

Figure S1. XRD patterns of CNSQ (above) and CCSQ-Tol (below) powder, the inset photo shows the two compounds in solid state. 10

Figure S2. a) ORTEP diagram of CNSQ, The dihedral angle between the dihydroxylphenyl and indolinyl is 4.0°, the C-N bond is calculated to be 1.38 Å b) Packing diagram of CNSQ. Hydrogen atoms are omitted for clarity. 11

Figure S3. TGA curves, tested under nitrogen atmosphere with heating rate of 10 °C per minute. 12

Figure S4. Absorption and emission spectra of two molecules in toluene. 13

Figure S5. a) Absorption of bland films, b) EQE curves and c) J-V curves of ASQs-based devices after thermal annealing treatment. 14

Figure S6. Pristine and blend films single carrier devices used for hole mobility measurement. 15

Figure S7. 3D image obtained by tapping-mode AFM showing the morphology of a) CNSQ: $PC_{71}BM$=1:5 (w/w) blend film and b) CCSQ-Tol: $PC_{71}BM$=1:5 (w/w) blend film, size 5×5 μm. Bright-field TEM images of c) CNSQ:$PC_{71}BM$(1:5) and d) CCSQ-Tol: $PC_{71}BM$(1:5) film. White regions represent the domain of ASQ and dark regions represent the domain of $PC_{71}BM$. 16

Figure S8. Water contact measurement for a) CNSQ, b) CCSQ-Tol and c) $PC_{71}BM$. 17

Figure S9. Molecular diagrams on ground state of a) CNSQ b) CCSQ and c) CCSQ-Tol at M06-2X/6-31++G(d,p) level 18

Figure S10 Total density of states (TDOS) partial DOS (PDOS) and overlap DOS(OPDOS) for CCSQ. 19

Figure S11 ESP for a) ASQ b) ASQ-F and c) ASQ-DF at vertical excited state 20

Table S1 The calculated absorption and emission spectra at B3lyp/6-31g(d) and M06-2X/6-31++G(d, p) level in chloroform* in comparision with experimental data 21

Table S2 Detail information of CNSQ single crystal. 22

Table S3 Annealed OSC performance data with blended ratio of ASQs:PC_{71}BM=1:5 (w/w). ..23

Table S4 Optical and electrochemical properties of two molecules..24

Table S5 Solvatochromism of absorption and emission spectra 25

Table S6 Extremes on electrostatic potentials for asymmetric squaraines derivatives. The values in parentheses are the extreme on Fragment1 without NH group. 26

Table S7 Positions and values of ESP minima and maxima at ground, vertical and optimized excited states 27

1 Experimental Information

1.1 Synthesis and Device fabrication

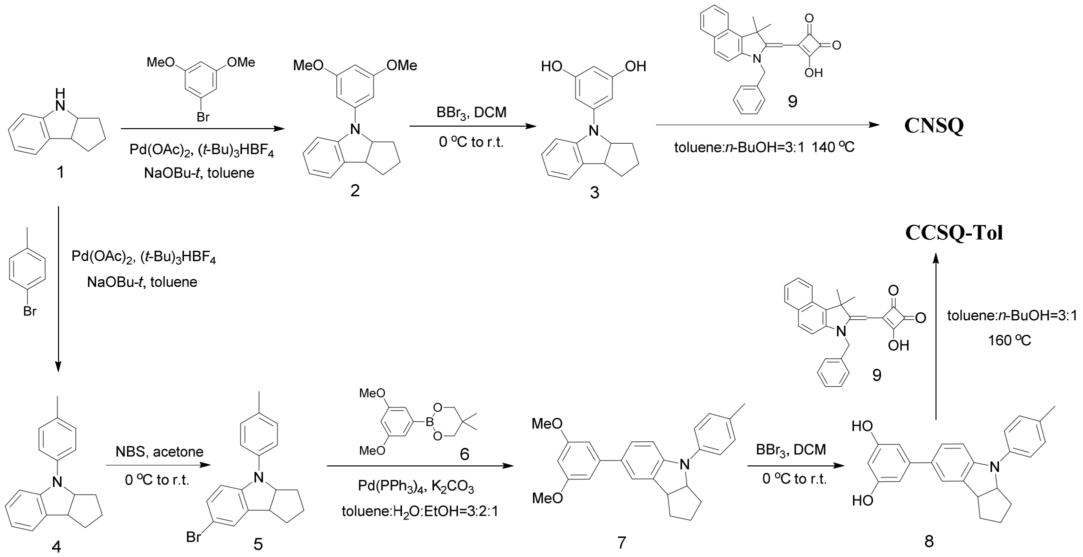

Scheme 1. Synthetic routes to the target molecules.

Synthetic routes to the target molecules are outlined in Scheme 1, intermediates compound 1, compound 6 and compound 9 were synthesized by modified procedures in reference.[1-9] The C-N or C-C linkage was formed *via* Buchwald-Hartwig or Suzuki-Miyaura[10] cross coupling reaction, respectively, followed by treatment with borontribromide to convert methoxy groups to hydroxy

groups. The compound 9 condensed with compound 3 or compound 8 in a toluene/*n*-BuOH mixture to obtain the target molecule CNSQ or CCSQ-Tol as illustrated in Scheme 1. CNSQ and CCSQ-Tol were then isolated with high (61%) and relatively low yields (12%), respectively. This could demonstrate the significant difference in reactivity between compound 3 and 8 should be originated from the different linkages. Both of target molecules exhibit good solubility in common-used solvents for solution process (*e. g.*, about 20 mg mL^{-1} in chloroform). Corresponding molecular structures were resolved using various spectroscopy instruments, e.g., ^{1}H NMR, ^{13}C NMR spectroscopy and HRMS.

Organic solar cells were fabricated with the following structure: ITO/MoO_3 (8 nm)/ASQ:PC71BM/LiF (7 Å)/Al. 190 nm thick indium-tin-oxide (ITO) coated substrate was pre-cleaned by sonicating in detergent, deionized water, acetone and ethanol respectively then exposed to UV/ozone for 5 min. Deposition of MoO3 on substrate was operated in a high vacuum chamber at the rate of 0.5 Å s^{-1} at the pressure of 3×10^{-4} Pa. The mixtures of squaraine donor material and PCBM in different ratio were spin-coated from 18 mg mL^{-1} chloroform solution on the MoO_3 layer at the rate of 1500 rmp for 30 s in glovebox. LiF and Al cathode were sequentially thermally deposited at the rate of 0.05 Å s^{-1} and 1.5 Å s^{-1}, respectively. Deposition rate and film thickness were in situ monitored by quartz crystal oscillator equipped on the substrate holder. EQE was obtained using light from EQE/IPCE Measurements Solar Cell Scan 100 (ZOLIX) system. Hole-only devices were fabricated with structure of ITO/MoO3 (8 nm)/ASQs:PC71BM (80 nm)/Au (100 nm).

1.2 Materials

8-(3,5-dimethoxyphenyl)-1,2,3,3a,8,8a-hexahydrocyclopenta[*a*]indene (2). A mixture of compound 1 (0.50 g, 3.1 mmol), 1-bromo-3,5-dimethoxybenzene (0.69 g, 3.1 mmol), $Pd(OAc)_2$ (palladium (II) acetate, 19 mg, 0.08 mmol), (t-Bu)$_3$PHBF_4 (tri-tert-butyl phosphine tetrafluoroborate, 44 mg, 0.15 mmol), NaOBu-*t* (sodium tert-butoxide, 0.40 g, 4 mmol) and toluene (70 mL) was refluxed for 12 h under argon atmosphere. The mixture was cooled down and filtered, the filtrate was concentrated *in vacuo* and purified by silica gel chromatography (eluent: hexane/ethyl acetate = 150/1) to afford a colourless oil. (0.61 g, 76%) ^{1}H NMR (400 MHz, $CDCl_3$, ppm) δ: 7.10 (d, J = 7.2 Hz, 1H, Ar-H), 7.08-7.02 (m, 2H, Ar-H), 6.73 (td, ^{3}J = 7.2 Hz, ^{4}J = 1.2 Hz, 1H, Ar-H), 6.46 (d, J =

2.0 Hz, 2H, Ar-H), 6.12 (t, J = 2.0 Hz, 1H, Ar-H), 4.70 (td, ^3J = 7.6Hz, ^4J = 2.4 Hz, N-CH-), 3.81 (t, J = 7.6 Hz, 1H, -CH-), 3.79 (s, 6H, $-OCH_3$), 2.08-1.55 (m, 6H, $-CH_2-$).

5-(1,2,3,3a,8,8a-hexahydrocyclopenta[*a*]inden-8-yl)benzene-1,3-diol (3). Compound 2 (0.51 g, 1.7 mmol) was dissolved in dichloromethane (25 mL), then solution of BBr_3 (borontribromide, 4.20 g, 17 mmol) in dichloromethane (30 mL) was added to reaction dropwise in ice bath, then the mixture was stirred at room temperature for 24 h. The solution was poured into icy water and extracted with dichloromethane for three times, then the combined organic phase was washed with water, saturated $NaHCO_3$ solution and water subsequently and dried with anhydrous $MgSO_4$. After filtration, the solution was concentrated *in vacuo* and purified by silica gel chromatography (eluent: dichloromethane/ethyl acetate = 9/1) to afford a white solid (0.41 g, 80%). ^{1}H NMR (400 MHz, $CDCl_3$, ppm) δ: 7.14-7.06 (m, 3H, Ar-H), 6.83-6.78 (m, 1H, Ar-H), 6.38 (s, 2H, Ar-H), 6.08 (s, 1H, Ar-H), 4.64-4.60 (m, 1H, -CH-), 3.87-3.78 (m, 1H, -CH-), 2.00-1.88 (m, 4H, $-CH_2-$), 1.68-1.60 (br, 1H, $-CH_2-$), 1.44-1.39 (br, 1H, $-CH_2-$).

4-(*p*-tolyl)-1,2,3,3a,4,8b-hexahydrocyclopenta[*b*]indole (4). A mixture of compound 1 (1.62 g, 0.95 mmol), 1-bromo-4-methylbenzene (1.51 g, 9.5 mmol), $Pd(OAc)_2$ (64 mg, 0.28 mmol), $(t\text{-Bu})_3PHBF_4$ (138 mg, 0.48 mmol), NaOBu-*t* (1.28 g, 13.3 mmol) and toluene (120 mL) was refluxed for 12 h under argon atmosphere. The mixture was cooled down and filtered, the filtrate was concentrated *in vacuo* and purified by silica gel chromatography (eluent: hexane/ethyl acetate = 150/1) to afford a colourless oil (1.65 g, 70%). ^{1}H NMR (400 MHz, $CDCl_3$, ppm) δ: 7.16 (s, 4H, Ar-H), 7.10 (d, J = 7.2 Hz, 1H, Ar-H), 7.03 (t, J = 8.0 Hz, 1H, Ar-H), 6.86 (d, J = 8.0 Hz, 1H, Ar-H), 6.68 (t, J = 7.6 Hz, 1H, Ar-H), 4.47 (td, ^3J = 7.2 Hz, ^4J = 2.0 Hz, 1H, N-CH), 3.78 (td, ^3J = 8.8 Hz, ^4J = 2.8 Hz, 1H, -CH-), 2.32 (s, 3H, $Ar-CH_3$), 2.17-1.60 (m, 6H, $-CH_2-$).

7-bromo-4-(*p*-tolyl)-1,2,3,3a,4,8b-hexahydrocyclopenta[*b*]indole (5). Compound 4 (1.60 g, 6.4 mmol) was dissolved in acetone (25 mL), NBS (*N*-bromosuccinimide, 1.14 g, 6.4 mmol) in acetone (35 mL) was added to reaction dropwise in ice bath and stirred at room temperature for 1 h. The resulting mixture was poured into water and extracted with dichloromethane. The combined

dichloromethane solution was concentrated *in vacuo* and recrystallized in hexane to afford a white crystal (1.66 g, 81%). ^{1}H NMR (400 MHz, $CDCl_3$, ppm) δ: 7.16 (s, 1H, Ar-H), 7.13 (s, 4H, Ar-H), 7.07 (dd, ^3J = 8.4 Hz, ^4J = 2.0 Hz, 1H, Ar-H), 6.71 (d, J = 8.4Hz, 1H, Ar-H), 4.47 (td, ^3J = 7.2 Hz, ^4J = 2.0 Hz, 1H, N-CH-), 3.78 (td, ^3J = 8.8 Hz, ^4J = 2.8 Hz, 1H, -CH-), 2.32 (s, 3H, Ar-CH_3), 2.17-1.60 (m, 6H, -CH_2-).

7-(3,5-dimethoxyphenyl)-4-(*p*-tolyl)-1,2,3,3a,4,8b-hexahydrocyclopenta[*b*]indole (7). A mixture of compound 5 (0.81 g, 3.2 mmol), compound 6 (0.82 g, 2.5 mmol), $Pd(PPh_3)_4$ [tetrakis(triphenylphosphine)palladium(0), 289 mg, 0.25 mmol), K_2CO_3 (1.38 g, 10 mmol), toluene (7.5 mL), deionized water (5.0 mL) and ethanol (2.5 mL) was degassed and heated to 110 ^oC for 24 h. The resulting mixture was concentrated and purified by silica gel chromatography (eluent: hexane/ethyl acetate = 150/1) to afford a colourless oil (0.69 g, 72%). ^{1}H NMR (400 MHz, $CDCl_3$, ppm) δ: 7.34 (s, 1H, Ar-H), 7.28 (d, J = 8.0 Hz, 1H, Ar-H), 7.20 (d, J = 8.8 Hz, 2H, Ar-H), 7.15 (d, J = 8.4 Hz, 2H, Ar-H), 6.93 (d, J = 8.4 Hz, 1H, Ar-H), 6.70 (s, 2H, Ar-H), 6.39 (s, 1H, Ar-H), 4.80 (t, J = 7.6 Hz, 1H, N-CH-), 3.86 (t, J = 8.4 Hz, 1H, -CH-), 3.84 (s, 6H, -OCH_3), 2.33 (s, 3H, -CH_3), 2.11-1.50 (m, 6H, -CH_2-). HRMS (ESI) *m/z* calcd for $[M + H]^+$: 386.2115, found: 386.2115.

5-(4-(*p*-tolyl)-1,2,3,3a,4,8b-hexahydrocyclopenta[*b*]indol-7-yl)benzene-1,3-diol (8). Compound 7 (0.69 g, 1.8 mmol) was dissolved in dichloromethane (20 mL), then the solution of BBr_3 (4.60 g, 18 mmol) in dichloromethane (30 mL) was added dropwise to reaction in ice bath and stirred at room temperature for 72 h. The solution was poured into icy water and extracted with dichloromethane for three times, the combined organic phase was washed with water, saturated $NaHCO_3$ solution, water subsequently and dried with anhydrous $MgSO_4$. After filtration, the solution was concentrated *in vacuo* and purified by silica gel chromatography (eluent: dichloromethane/ethyl acetate = 9/1) to afford a white solid (0.57 g, 89%). ^{1}H NMR (400 MHz, DMSO, ppm) δ: 9.18 (s, 2H, -OH), 7.28 (s, 1H, Ar-H), 7.22-7.14 (m, 5H, Ar-H), 6.88 (d, J = 8.0 Hz, 1H, Ar-H), 6.40 (s, 2H, Ar-H), 6.12 (s, 1H, Ar-H), 4.83 (t, J = 7.2 Hz, 1H, N-CH-), 3.83 (t, J = 7.6 Hz, 1H, -CH=), 2.28 (s, 3H, -CH_3), 2.10-1.55 (m, 6H, -CH_2-)

2-((3-benzyl-1,1-dimethyl-1*H*-benzo[e]indol-2(3*H*)-ylidene)methyl)-4-(2,6-dihydroxy-4-(1,3,3a,8b-tetrahydrocyclopenta[b]indol-4(2*H*)-yl)phenyl)cyclobuta-1,3-diene-1,3-bis(olate) (CNSQ). A mixture of compound 9 (0.40 g, 0.1 mmol), compound 3 (0.27 g, 0.1 mmol), toluene (15 mL) and *n*-butanol (5 mL) was refluxed for 24 h. After the mixture was cooled down, the solvents were removed *in vacuo*. The crude product was purified by silica gel chromatography (eluent: dichloromethane) to afford a golden solid, then recrystallized from dichloromethane/methanol (10:1, v/v) to give a metallic golden crystal (0.39 g, 61%). ^{1}H NMR (400 MHz, $CDCl_3$, ppm) δ: 12.40 (br, 2H, -OH), 8.24 (d, *J* = 8.4Hz, 1H, Ar-H), 7.92 (d, *J* = 8.4 Hz, 1H, Ar-H), 7.86 (d, *J* = 8.4 Hz, 1H, Ar-H), 7.64 (td, ^3J = 6.8 Hz, ^4J = 1.2 Hz, 1H, Ar-H), 7.49 (t, *J* = 7.2 Hz, 1H, Ar-H), 7.38-7.28 (m, 5H, Ar-H), 7.22-7.14 (m, 4H, Ar-H), 6.94 (t, *J* = 7.6 Hz, 1H, Ar-H), 6.34 (s, 2H, Ar-H), 6.02 (s, 1H, -CH=), 5.43 (s, 2H, $-CH_2-$), 4.68 (td, ^3J = 8.8 Hz, ^4J = 2.8 Hz, 1H, N-CH-), 3.90 (t, *J* = 7.2 Hz, 1H, -CH-), 2.11 (s, 6H, $-CH_3$), 2.04-1.37 (m, 6H, $-CH_2-$). ^{13}C NMR (100 MHz, $CDCl_3$, ppm) δ: 174.38, 169.71, 168.53, 162.00, 151.81, 142.93, 138.00, 135.92, 134.33, 132.25, 130.96, 129.28, 128.82, 128.41, 127.48, 127.32, 126.76, 126.38, 125.12, 124.32, 123.82, 121.68, 121.42, 112.70, 109.68, 103.89, 95.72, 86.73, 67.65, 51.15, 46.73, 44.47, 33.61, 32.75, 25.68, 23.30. HRMS (ESI) *m/z* calcd for $[M + H]^+$: 645.2759, found: 645.2759.

2-((3-benzyl-1,1-dimethyl-1*H*-benzo[*e*]indol-2(3*H*)-ylidene)methyl)-4-(2,6-dihydroxy-4-(4-(*p*-tolyl)-1,2,3,3a,4,8b-hexahydrocyclopenta[*b*]indol-7-yl)phenyl)cyclobuta-1,3-diene-1,3-bis(olate) (CCSQ-Tol). A mixture of compound 9 (107 mg, 0.3 mmol), compound 8 (118 mg, 0.3 mmol), toluene (7.5 mL) and *n*-butanol (2.5 mL) was heated to 160 °C and reacted for 36 h. After reaction was cooled down, the solvent was removed *in vacuo*. The crude product was purified by silica gel chromatography (eluent: dichloromethane) to afford a blue solid, then recrystallized from dichloromethane /methanol (10:1, v/v) to give a blue solid (27 mg, 12%). ^{1}H NMR (400 MHz, $CDCl_3$, ppm) δ: 12.07 (s, 1H, -OH), 11.95 (s, 1H, -OH), 8.25 (d, *J* = 8.4Hz, 1H, Ar-H), 7.95 (d, *J* = 8.0 Hz, 1H, Ar-H), 7.90 (d, *J* = 9.2 Hz, 1H, Ar-H), 7.68 (t, *J* = 7.2 Hz, 1H, Ar-H), 7.55 (t, *J* = 7.6 Hz, 1H, Ar-H), 7.46 (s, 1H, Ar-H), 7.42-7.33 (m, 5H, Ar-H), 7.23-7.15 (m, 6H, Ar-H), 6.90 (d, *J* = 7.6 Hz, 1H, Ar-H), 6.68 (s, 1H, Ar-H), 6.65 (s, 1H, Ar-H), 6.21 (s, 1H, -CH=), 5.53 (s, 2H, $-CH_2-$), 4.85 (br, 1H, N-CH-), 3.86 (br, 1H, -CH-), 2.34 (s, 3H, $-CH_3$), 2.13 (s, 6H, $-CH_3$), 2.10-1.47 (m, 6H, $-CH_2-$). ^{13}C NMR (100 MHz, $CDCl_3$, ppm) δ: 184.05, 181.01, 178.23, 174.00, 161.62, 161.13, 149.15, 139.83, 138.51, 136.68, 135.52, 132.64, 132.49, 131.93, 130.65, 129.92, 129.82, 129.57, 128.95, 128.80,

128.09, 126.76, 126.15, 126.09, 123.29, 122.85, 120.45, 111.00, 108.13, 107.33, 106.02, 105.85, 89.39, 69.34, 53.04, 48.40, 45.27, 35.10, 33.62, 28.39, 24.40, 20.83. HRMS (ESI) *m/z* calcd for $[M + H]^+$: 735.3228, found: 735.3226.

1.3 Instruments and measurements

The ^1H and ^{13}C NMR spectra were recorded on a Bruker Avance AVII-400 Spectrometer. Chemical shift values (δ) were expressed in parts per million (ppm) using tetramethylsilane (TMS) as internal standard. High resolution mass (HRMS) spectra were measured on a Shimadzu LCMS-IT-TOF. Thermogravimetric analysis (TGA) was conducted on a Perkin Elmer TGA Q500 instrument with a heating rate of 10 °C min^{-1} under nitrogen atmosphere. UV−Vis-NIR spectra of target molecules were obtained with a Lambda 950 scanning spectrophotometer. Solution samples were prepared in chloroform solution at a concentration of 2.00×10^{-6} M and film samples were spin-coated from chloroform solution (5.0 mg mL^{-1}, 1500 rpm, 30 s) on quartz substrates. The photoluminescence (PL) spectra of solution samples (2.0×10^{-6} M) were recorded on a PerkinElmer LS55 fluorescence spectrophotometer at room temperature. Photoluminescence quantum yield (PLQY) in toluene was determined using 2,4-bis[4-(N,N-diisobutylamino)-2,6-dihydroxyphenyl] squaraine in 1.0×10^{-6} $mol \cdot L^{-1}$ (φ = 0.80) as standard under excitation of 600 nm. The transient photoluminescence decay characteristics of the predegassed solution samples were recorded on a Single Photon Counting Controller FluoroHub-B. Cyclic voltammetry (CV) experiments were performed in 2.50×10^{-4} M degassed anhydrous dichloromethane solution with tetrabutylammonium perchlorate (Bu_4NClO_4, 0.1 M) at a scan rate of 50 mV s^{-1} with a LK 2010 electrochemical workstation. All measurements were carried out under argon atmosphere with a conventional three-electrode system employing a Pt disk, a Pt wire and a $Ag/AgNO_3$ (0.1 M in acetonitrile) electrode as the working electrode, counter electrode and reference electrode, respectively. The potentials were measured against an Ag/Ag^+ reference electrode and reported with reference to the ferrocene/ferrocenium (Fc/Fc^+) redox couple. Atomic force microscopy (AFM) in the tapping mode was used to characterize the surface morphology of the samples using an MFP 3D Asylum Research instrument. Single crystal X-ray diffraction data of the target molecules were obtained on a Xcalibur E X-ray single crystal diffractometer equipped with graphite monochromator Mo-Ka (λ = 0.71073 Å) radiation. The powder X-ray diffraction spectroscopy (XRD) patterns were captured by a Tongda TD-3500. X-ray power diffractometer with Cu-Kα radiation (λ = 0.15148 nm) operating at 30.0 kV and 20.0 mA. The transmission electron microscopy (TEM) investigation was performed on FEI Tecnai G^2F20. The

samples for TEM measurement were prepared by spin casting the ASQ:$PC_{71}PM$ solution on glass/PEDOT:PSS substrate, then floating the film on deionized water surface, and transferring to 200-mesh copper TEM grids. Water contact measurements were performed by the static drop method on a JC2000C1 Contact Angle Measuring Instrument.

1. L. Yang, Q. Yang, D. Yang, Q. Luo, Y. Zhu, Y. Huang, Z. Lu and S. Zhao, *J. Mater. Chem. A*, 2014, **2**, 18313–18321.
2. J. H. Yum, P. Walter, S. Huber, D. Rentsch, T. Geiger, F. Nuesch, F. De Angelis, M. Gratzel and M. K. Nazeeruddin, *J. Am. Chem. Soc.*, 2007, **129**, 10320-10321.
3. M. Akhtaruzzaman, S. Yohei, N. Asao, A. Islam, E. Kwon, A. ElShafei, L. Han and Y. Yamamoto, *J. Mater. Chem.*, 2012, **22**, 10771.
4. C. G. Gordon, J. L. Mackey, J. C. Jewett, E. M. Sletten, K. N. Houk and C. R. Bertozzi, *J. Am. Chem. Soc.*, 2012, **134**, 9199-9208.
5. J. D. Knight and D. M. Coltart, *Tetrahedron Lett.*, 2013, **54**, 5470-5472.
6. V. Buinauskaitė, V. Martynaitis, S. Mangelinckx, G. Kreiza, N. De Kimpe and A. Šačkus, *Tetrahedron*, 2012, **68**, 9260-9266.
7. G. S. Welmaker and J. E. Sabalski, *Tetrahedron Lett.*, 2004, **45**, 4851-4854.
8. S. Fuse, S. Sugiyama and T. Takahashi, *Chem.-Asian J.*, 2010, **5**, 2459-2462.
9. Q. Nguyen, K. Sun and T. G. Driver, *J. Am. Chem. Soc.*, 2012, **134**, 7262-7265.

2 Supplementary Figures and Tables

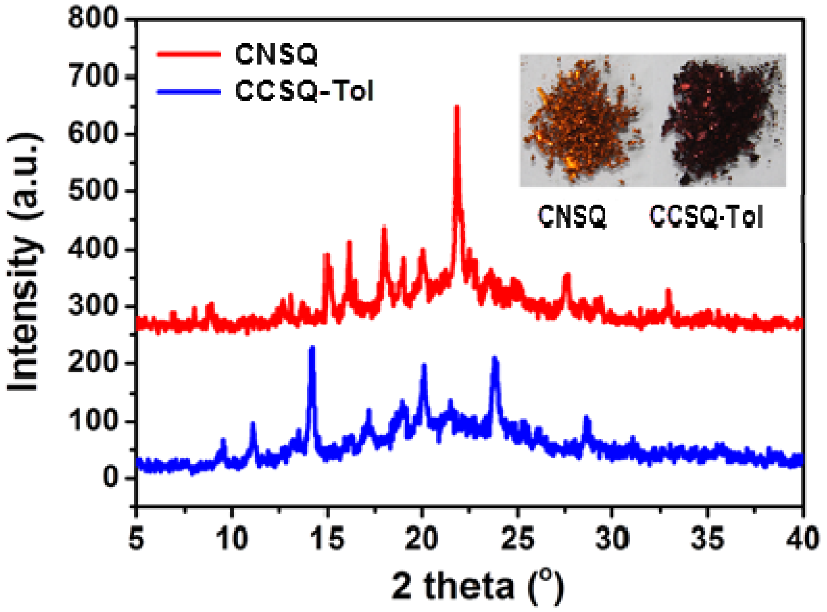

Figure S1. XRD patterns of CNSQ (above) and CCSQ-Tol (below) powder, the inset photo shows the two compounds in solid state.

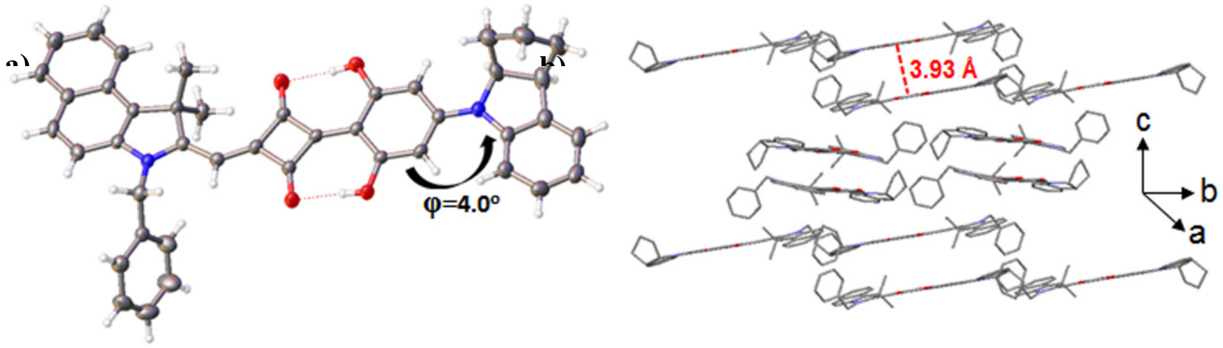

Figure S2. a) ORTEP diagram of CNSQ, The dihedral angle between the dihydroxylphenyl and indolinyl is 4.0°, the C-N bond is calculated to be 1.38 Å b) Packing diagram of CNSQ. Hydrogen atoms are omitted for clarity.

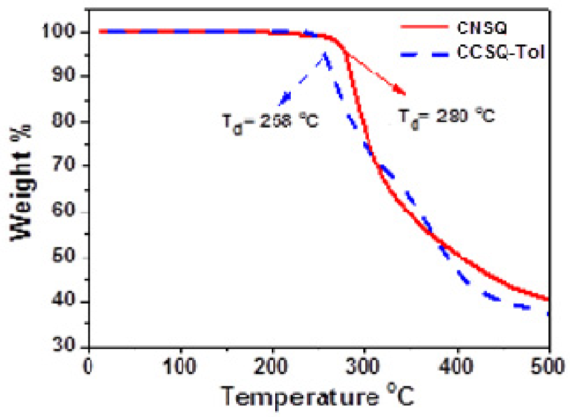

Figure S3. TGA curves, tested under nitrogen atmosphere with heating rate of 10 °C per minute**.**

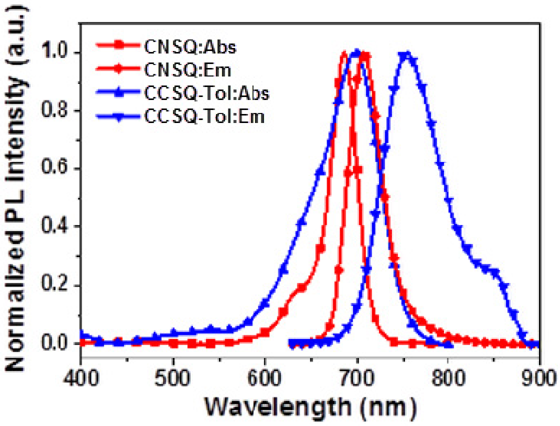

Figure S4. Absorption and emission spectra of two molecules in toluene.

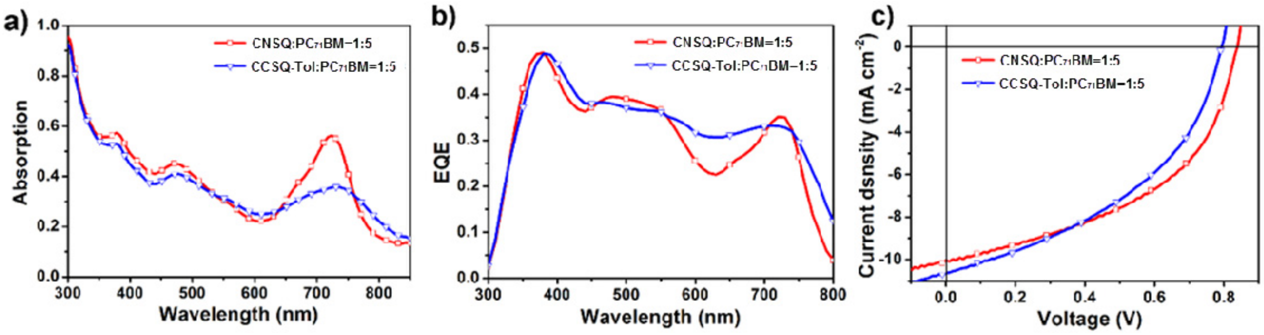

Figure S5. a) Absorption of bland films, b) EQE curves and c) J-V curves of ASQs-based devices after thermal annealing treatment.

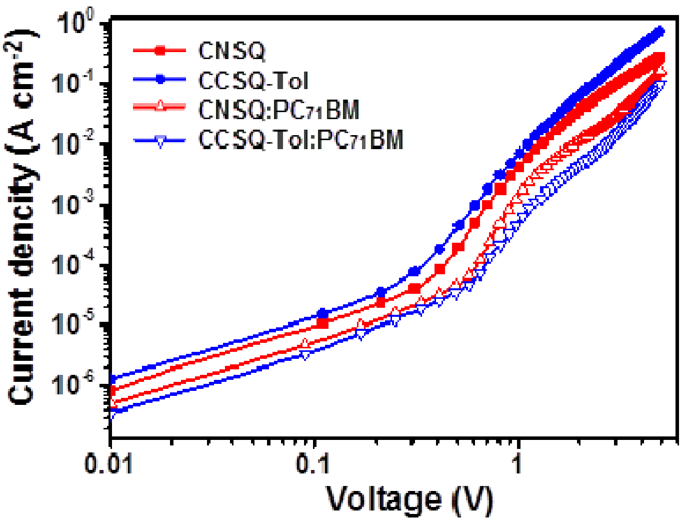

Figure S6. Pristine and blend films single carrier devices used for hole mobility measurement.

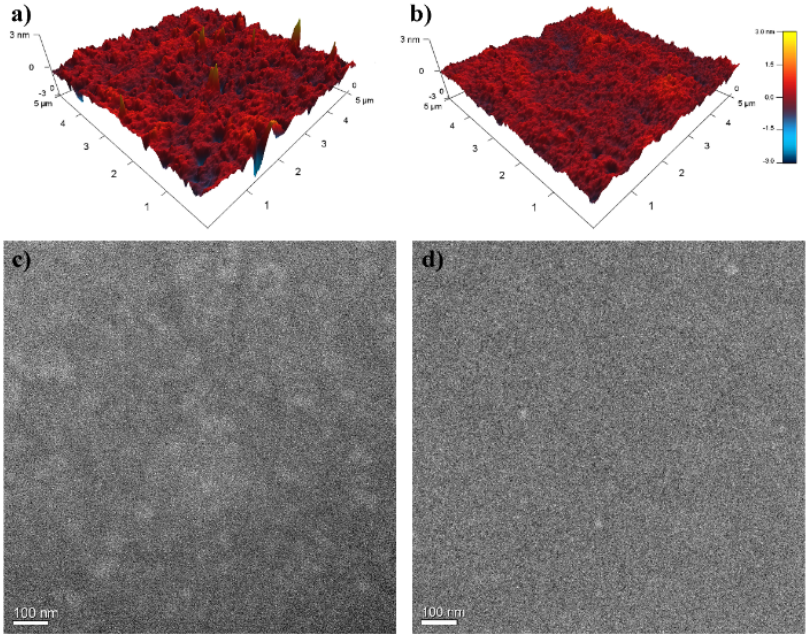

Figure S7. 3D image obtained by tapping-mode AFM showing the morphology of a) CNSQ: $PC_{71}BM$=1:5 (w/w) blend film and b) CCSQ-Tol: $PC_{71}BM$=1:5 (w/w) blend film, size 5×5 μm. Bright-field TEM images of c) CNSQ:$PC_{71}BM$(1:5) and d) CCSQ-Tol: $PC_{71}BM$(1:5) film. White regions represent the domain of ASQ and dark regions represent the domain of $PC_{71}BM$.

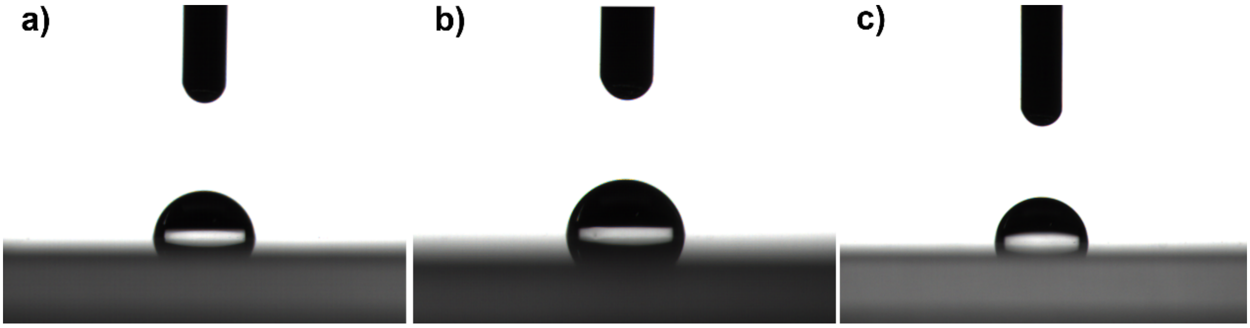

Figure S8. Water contact measurement for a) CNSQ, b) CCSQ-Tol and c) $PC_{71}BM$.

a)

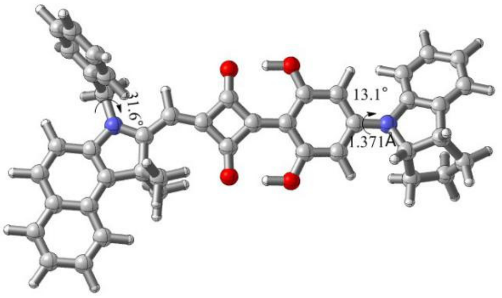

b)

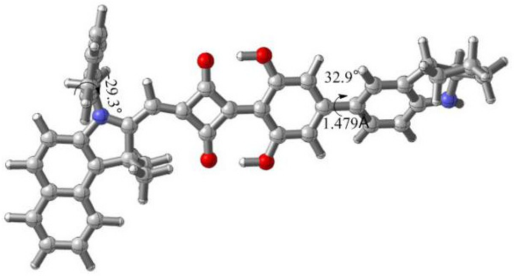

c)

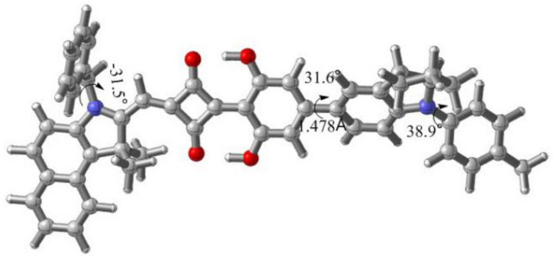

Figure S9. Molecular diagrams on ground state of a) CNSQ b) CCSQ and c) CCSQ-Tol at M06-2X/6-31++G(d,p) level

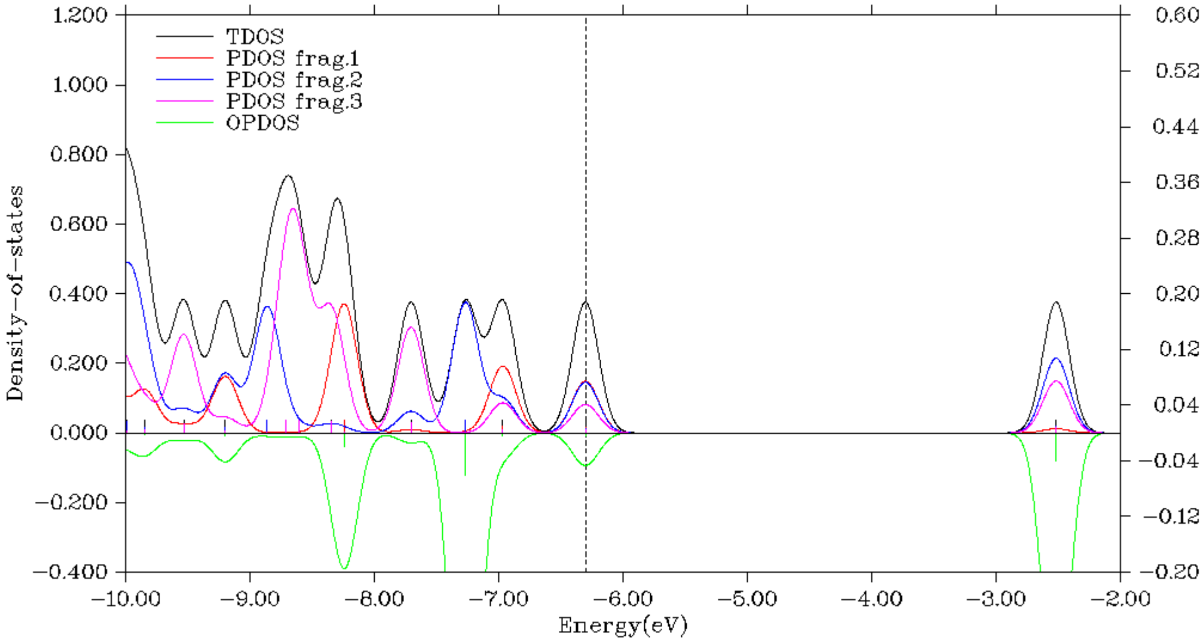

Figure S10 Total density of states (TDOS) partial DOS (PDOS) and overlap DOS(OPDOS) for CCSQ.

a)

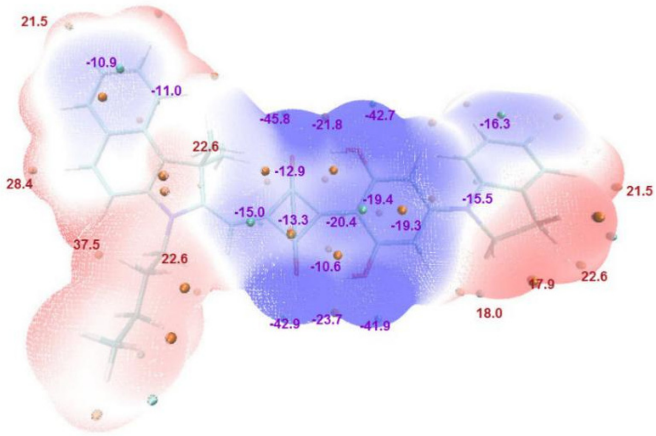

b)

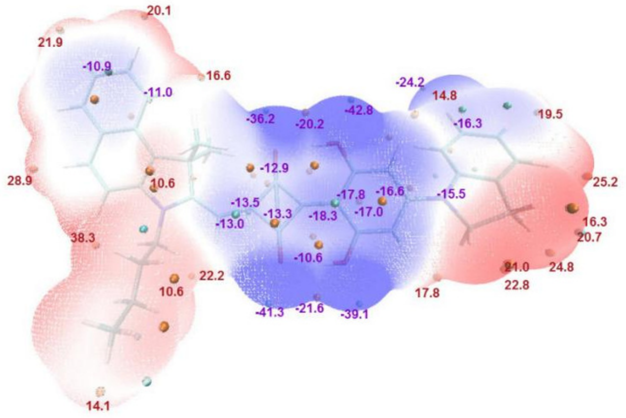

c)

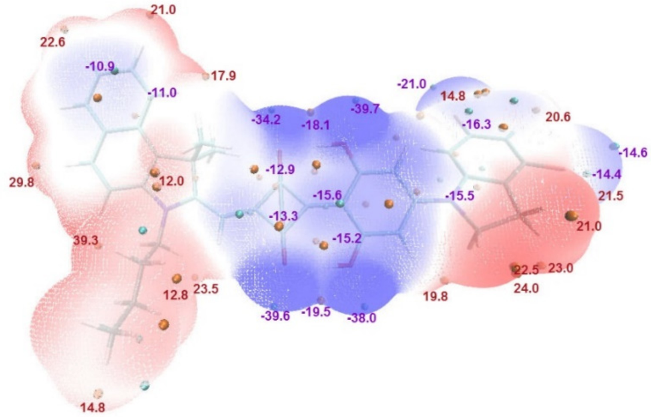

Figure S11 ESP for a) ASQ b) ASQ-F and c) ASQ-DF at vertical excited state

Table S1 The calculated absorption and emission spectra at B3lyp/6-31g(d) and M06-2X/6-31++G(d, p) level in chloroform* in comparision with experimental data

		CNSQ		CCSQ-Tol		CCSQ	
		S_0	S_1	S_0	S_1	S_0	S_1
B3lyp/6-31g(d)	φ_1(°)	22.8	27.4	28.4	38.1	29.2	37.7
	B_{12}(Å)	1.380	1.399	1.475	1.485	1.475	1.484
M06-2X/6-31++G(d, p)	φ_1(°)	13.1	15.8	31.6	23.6	32.9	23.7
	B_{12}(Å)	1.371	1.371	1.478	1.457	1.479	1.458

* φ_1 is defined as the torsion angle between Fragment 1 and Fragment 2; $B_{23,}$ the covalent bond distance between Fragment 1 and Fragment 2; *f,* the oscillator strength; *ε,* molar extinction coefficient.

Table S2 Detail information of CNSQ single crystal**.**

	CNSQ		CNSQ
Empirical formula	$C_{41}H_{37}N_5O_3$	F(000)	1368.0
Formula weight	647.76	2*Θ* range for data collection	6 to 58°
Temperature/K	142.95(10)	Index ranges	$-18 \le h \le 18$, $-20 \le k \le 20$, $-21 \le l \le 18$
Crystal system	monoclinic		
Space group	$P2_1/n$		
a/Å	13.7967(7)	Reflections collected	17523
b/Å	15.4496(7)	Independent reflections	7394[R(int) = 0.0258]
c/Å	16.2496(8)	Data/restraints/parameters	7394/0/444
α/°	90.00	Goodness-of-fit on F^2	1.033
β/°	111.692(6)	Final R indexes [I>=2σ (I)]	R1=0.0784 wR2= 0.2165
γ/°	90.00		
Volume/Å^3	3218.4(3)	Final R indexes [all data]	R1=0.1118 wR2= 0.2411
Z	4		
ρ_{calc} mg/mm^3	1.337	Largest diff. peak/hole/eÅ^{-3}	0.58/-0.62
μ/mm^{-1}	0.086		

Table S3 Annealed OSC performance data with blended ratio of ASQs:$PC_{71}BM$=1:5 (w/w).

Active layer (w/w) [a]	J_{sc} (mA cm^{-2})	V_{oc} (V)	FF	PCE [b] (%)
CNSQ:$PC_{71}BM$=1:5	10.03	0.84	0.48	4.02 (3.84)
CCSQ-Tol:$PC_{71}BM$=1:5	10.64	0.79	0.43	3.63 (3.45)

[a] Anneal at 90 °C for 10min. [b] report as the form of best value (average value), average value obtained from at least 16 devices.

Table S4 Optical and electrochemical properties of two molecules

Compound	Absorption in $CHCl_3$			Absorption in film				
	λ_{max} (nm)	FWHM (nm)	log ε [a]	λ_{max} (nm)	FWHM (nm)	ΔE_g^{opt} (eV)	HOMO (eV)	LUMO (eV) [b]
CNSQ	686	38	5.36	729	153	1.53	-5.10	-3.57
CCSQ-Tol	690	108	5.10	706	195	1.48	-5.04	-3.56

[a] ε: molar extinction coefficient. [b] LUMO=HOMO+ΔE_g^{opt}

Table S5 Solvatochromism of absorption and emission spectra

Abs.	Hex	Tol	CB	CF	DCM	ACN
LQ-5	674	686	689	685	682	670
LQ-7	692	698	694	691	680	648
Em.	Hex	Tol	CB	CF	DCM	ACN
LQ-5	686	711	716	714	713	706
LQ-7	712	753	790	790	809	658

Table S6 Extremes on electrostatic potentials for asymmetric squaraines derivatives. The values in parentheses are the extreme on Fragment1 without NH group.

Molecules	Ground state	Vertical excited state			Optimized excited state
	Extremes[a]	Extremes[a]	T_e[b]	T_h[c]	Extremes[a]
CNSQ	37.4, -45.6, 18.0	34.2, -45.8, 21.7	79.8	83.2	34.5, -46.3, 21.8
CCSQ-Tol	44.1, -47.0, 17.8	36.5, -46.2, 25.6	83.2	90.3	35.3, -48.8, 28.3
CCSQ	43.7, -47.4, 39.1(12.0)	37.6, -44.9, 46.5(16.7)	85.0	88.6	36.0, -48.3, 54.3(20.8)
ASQ5	41.8, -44.0, 17.8	37.5, -42.9, 22.6	79.3	84.7	37.9, -43.2, 22.6
ASQ5-F	43.2, -44.1, 20.4	38.3, -42.1, 25.2	82.4	85.3	38.7, -42.7, 25.2
ASQ5-DF	44.6, -42.4, 17.7	39.3, -39.7, 24.0	82.1	84.3	39.8, -40.5, 24.0
CCSQ'-Ox	46.5, -46.4, 13.3	40.2, -41.7, 18.5	86.6	88.2	40.4, -42.8, 18.0

[a]Extremes are given in the sequence from the fragments 1 to 3 of asymmetric squaraines.

[b]T_e, the difference of ESP for electron transfer;

[c]T_h the difference of ESP for hole transfer

Table S7 Positions and values of ESP minima and maxima at ground, vertical and optimized excited states

CNSQ-SCF.pdb

HETATM	10	O	MOL	A	1	-0.194	4.400	-0.683	1.00	-45.59
HETATM	13	O	MOL	A	1	3.538	4.208	-0.139	1.00	-45.47
HETATM	11	O	MOL	A	1	2.644	-3.996	0.240	1.00	-41.12
HETATM	9	O	MOL	A	1	-0.627	-3.488	0.172	1.00	-35.26
HETATM	20	C	MOL	A	1	1.742	4.034	-0.634	1.00	-25.7
HETATM	15	O	MOL	A	1	4.060	-1.435	-1.908	1.00	-24.66
HETATM	14	O	MOL	A	1	3.708	-0.634	2.123	1.00	-23.54
HETATM	12	O	MOL	A	1	3.045	-0.090	-2.095	1.00	-22.37
HETATM	21	C	MOL	A	1	3.704	-0.231	-1.836	1.00	-21.84
HETATM	18	O	MOL	A	1	7.940	3.076	0.800	1.00	-21.81
HETATM	16	C	MOL	A	1	0.826	-3.397	0.277	1.00	-20.39
HETATM	20	O	MOL	A	1	9.229	0.774	-2.163	1.00	-19.04
HETATM	17	O	MOL	A	1	6.196	-0.634	-1.745	1.00	-14.05
HETATM	7	O	MOL	A	1	-3.784	4.719	1.330	1.00	-12.36
HETATM	19	C	MOL	A	1	1.586	1.490	-1.915	1.00	-10.95
HETATM	18	C	MOL	A	1	1.193	-1.285	-1.579	1.00	-8.89
HETATM	5	O	MOL	A	1	-6.630	-3.564	2.328	1.00	-8.74
HETATM	6	O	MOL	A	1	-6.466	-4.578	-1.577	1.00	-8.59

HETATM	17	C	MOL A	1	1.030	-0.905	1.418	1.00		-8.5
HETATM	1	O	MOL A	1	-7.826	-3.411	2.224	1.00		-8.42
HETATM	2	O	MOL A	1	-7.690	-4.469	-1.603	1.00		-8.18
HETATM	8	O	MOL A	1	-1.482	0.951	-2.543	1.00		-7.97
HETATM	4	O	MOL A	1	-7.168	2.674	2.109	1.00		-7.88
HETATM	4	C	MOL A	1	-7.123	-2.725	1.889	1.00		-5.83
HETATM	6	C	MOL A	1	-7.054	-3.549	-1.645	1.00		-5.45
HETATM	3	O	MOL A	1	-7.570	0.024	1.418	1.00		-5.09
HETATM	16	O	MOL A	1	5.692	-3.800	-0.785	1.00		-2.13
HETATM	8	C	MOL A	1	-6.718	-0.737	1.370	1.00		-1.92
HETATM	15	C	MOL A	1	-1.186	-1.015	-2.119	1.00		-1.51
HETATM	19	O	MOL A	1	8.544	-2.930	-2.374	1.00		-1.28
HETATM	14	C	MOL A	1	-1.229	-0.368	1.708	1.00		-0.17
HETATM	30	C	MOL A	1	9.595	-1.614	-2.434	1.00		2.32
HETATM	22	C	MOL A	1	6.451	-2.887	-2.292	1.00		2.77
HETATM	23	C	MOL A	1	6.511	2.966	-2.480	1.00		4.38
HETATM	29	C	MOL A	1	9.633	-4.892	-0.925	1.00		11.24
HETATM	27	C	MOL A	1	8.989	4.398	-2.503	1.00		11.49
HETATM	24	C	MOL A	1	7.167	-4.596	1.380	1.00		11.94
HETATM	28	C	MOL A	1	9.259	-3.934	2.599	1.00		13.75

HETATM	25	C	MOL	A	1	7.685	-2.849	2.248	1.00	13.86
HETATM	32	C	MOL	A	1	11.985	3.333	-0.556	1.00	13.86
HETATM	26	C	MOL	A	1	8.767	-0.991	3.763	1.00	14.23
HETATM	11	C	MOL	A	1	-4.389	-0.495	-2.560	1.00	16.2
HETATM	31	C	MOL	A	1	11.879	0.162	1.569	1.00	17.99
HETATM	12	C	MOL	A	1	-4.035	0.896	2.192	1.00	19.03
HETATM	10	C	MOL	A	1	-4.644	3.341	4.499	1.00	19.54
HETATM	13	C	MOL	A	1	-3.552	-4.596	0.546	1.00	19.62
HETATM	7	C	MOL	A	1	-7.007	6.281	3.725	1.00	21.39
HETATM	9	C	MOL	A	1	-6.018	-6.977	1.134	1.00	21.69
HETATM	2	C	MOL	A	1	-9.635	-6.306	0.818	1.00	22.86
HETATM	3	C	MOL	A	1	-8.255	6.782	0.119	1.00	24.12
HETATM	1	C	MOL	A	1	-10.508	-0.641	-0.556	1.00	28.75
HETATM	5	C	MOL	A	1	-7.170	2.409	-2.390	1.00	37.4

CNSQ-CI.pdb

HETATM	9	O	MOL	A	1	-0.281	4.378	-0.792	1.00	-45.82
HETATM	12	O	MOL	A	1	3.504	4.202	-0.023	1.00	-44.97
HETATM	10	O	MOL	A	1	2.644	-3.998	0.108	1.00	-41.01
HETATM	8	O	MOL	A	1	-0.690	-3.463	0.257	1.00	-36.39
HETATM	23	C	MOL	A	1	1.742	4.034	-0.634	1.00	-25.93

HETATM	18	C	MOL A	1	0.826	-3.397	0.277	1.00		-21.11
HETATM	11	O	MOL A	1	2.675	-0.041	-2.185	1.00		-20.94
HETATM	24	C	MOL A	1	3.456	0.181	1.752	1.00		-20.35
HETATM	25	C	MOL A	1	3.704	-0.231	-1.836	1.00		-20.17
HETATM	14	O	MOL A	1	7.923	3.185	0.743	1.00		-17.15
HETATM	21	C	MOL A	1	1.328	1.897	1.070	1.00		-14.83
HETATM	6	O	MOL A	1	-3.745	4.659	1.302	1.00		-14.56
HETATM	7	O	MOL A	1	-1.194	0.562	-2.502	1.00		-14.53
HETATM	16	O	MOL A	1	9.155	0.878	-2.324	1.00		-14.24
HETATM	22	C	MOL A	1	1.693	1.475	-1.896	1.00		-14.13
HETATM	20	C	MOL A	1	1.219	-1.366	-1.526	1.00		-12.32
HETATM	19	C	MOL A	1	1.030	-0.905	1.418	1.00		-11.9
HETATM	4	O	MOL A	1	-6.590	-3.489	2.305	1.00		-10.9
HETATM	17	C	MOL A	1	0.009	0.825	-2.374	1.00		-10.75
HETATM	5	O	MOL A	1	-6.466	-4.578	-1.577	1.00		-10.75
HETATM	3	O	MOL A	1	-7.022	2.413	1.986	1.00		-10.45
HETATM	1	O	MOL A	1	-7.826	-3.411	2.224	1.00		-10.3
HETATM	4	C	MOL A	1	-7.123	-2.725	1.889	1.00		-8.13
HETATM	6	C	MOL A	1	-7.054	-3.549	-1.645	1.00		-7.75
HETATM	2	O	MOL A	1	-7.441	0.102	1.398	1.00		-7.75

HETATM	15	C	MOL	A	1	-1.300	-1.406	-2.384	1.00	-7.22
HETATM	16	C	MOL	A	1	-1.235	-0.520	1.824	1.00	-5.73
HETATM	8	C	MOL	A	1	-6.718	-0.737	1.370	1.00	-4.64
HETATM	13	O	MOL	A	1	5.692	-3.800	-0.785	1.00	0.83
HETATM	15	O	MOL	A	1	8.715	-3.023	-2.382	1.00	1.96
HETATM	26	C	MOL	A	1	6.451	-2.887	-2.292	1.00	5.87
HETATM	32	C	MOL	A	1	9.524	-1.553	-2.446	1.00	5.88
HETATM	11	C	MOL	A	1	-4.296	-1.288	-3.361	1.00	10.64
HETATM	33	C	MOL	A	1	9.621	-4.910	-0.797	1.00	13.97
HETATM	12	C	MOL	A	1	-3.976	0.979	2.243	1.00	14.29
HETATM	30	C	MOL	A	1	8.989	4.398	-2.503	1.00	14.71
HETATM	27	C	MOL	A	1	7.286	-4.475	1.490	1.00	15.08
HETATM	14	C	MOL	A	1	-3.574	-4.595	0.669	1.00	16.75
HETATM	31	C	MOL	A	1	9.228	-3.871	2.653	1.00	16.75
HETATM	35	C	MOL	A	1	11.985	3.333	-0.556	1.00	17.42
HETATM	10	C	MOL	A	1	-4.644	3.341	4.499	1.00	17.59
HETATM	29	C	MOL	A	1	8.767	-0.991	3.763	1.00	17.89
HETATM	28	C	MOL	A	1	7.685	-2.849	2.248	1.00	17.98
HETATM	7	C	MOL	A	1	-7.007	6.281	3.725	1.00	19.9
HETATM	9	C	MOL	A	1	-6.018	-6.977	1.134	1.00	20.17
HETATM	2	C	MOL	A	1	-9.635	-6.306	0.818	1.00	21.58

HETATM 34 C MOL A 1 11.799 0.052 1.607 1.00 21.71

HETATM 3 C MOL A 1 -8.255 6.782 0.119 1.00 22.56

HETATM 13 C MOL A 1 -3.989 3.429 -2.771 1.00 22.6

HETATM 1 C MOL A 1 -10.512 -0.774 -0.558 1.00 27.03

HETATM 5 C MOL A 1 -7.170 2.409 -2.390 1.00 34.24

CNSQ-CIOPT.pdb

HETATM 9 O MOL A 1 -0.185 4.400 -0.654 1.00 -46.28

HETATM 13 O MOL A 1 3.503 4.222 -0.048 1.00 -45.23

HETATM 12 O MOL A 1 2.641 -4.004 0.175 1.00 -41.13

HETATM 8 O MOL A 1 -0.674 -3.480 0.172 1.00 -36.81

HETATM 22 C MOL A 1 1.754 4.017 -0.705 1.00 -26.61

HETATM 17 C MOL A 1 0.868 -3.417 0.137 1.00 -21.61

HETATM 11 O MOL A 1 2.221 0.595 1.865 1.00 -21.36

HETATM 10 O MOL A 1 2.362 0.067 -2.206 1.00 -21.24

HETATM 23 C MOL A 1 3.420 0.200 1.765 1.00 -20.34

HETATM 24 C MOL A 1 3.824 -0.193 -1.801 1.00 -20.02

HETATM 15 O MOL A 1 7.994 3.164 0.746 1.00 -17.2

HETATM 20 C MOL A 1 1.424 1.920 1.075 1.00 -15.33

HETATM 6 O MOL A 1 -3.698 4.596 1.230 1.00 -14.91

HETATM	7	O	MOL A	1	-1.236	0.576	-2.499	1.00	-14.61
HETATM	21	C	MOL A	1	1.676	1.636	-1.840	1.00	-14.52
HETATM	17	O	MOL A	1	9.103	0.860	-2.363	1.00	-14.36
HETATM	19	C	MOL A	1	1.308	-1.353	-1.521	1.00	-12.6
HETATM	18	C	MOL A	1	1.073	-1.011	1.393	1.00	-12.24
HETATM	16	C	MOL A	1	-0.048	0.843	-2.367	1.00	-11.31
HETATM	4	O	MOL A	1	-6.628	-3.467	2.317	1.00	-10.84
HETATM	5	O	MOL A	1	-6.462	-4.518	-1.576	1.00	-10.67
HETATM	3	O	MOL A	1	-7.022	2.451	2.017	1.00	-10.41
HETATM	1	O	MOL A	1	-7.746	-3.401	2.212	1.00	-10.22
HETATM	4	C	MOL A	1	-7.164	-2.711	1.895	1.00	-8.05
HETATM	6	C	MOL A	1	-7.097	-3.535	-1.641	1.00	-7.68
HETATM	2	O	MOL A	1	-7.554	0.110	1.396	1.00	-7.61
HETATM	14	C	MOL A	1	-1.435	-1.476	-2.502	1.00	-7.3
HETATM	15	C	MOL A	1	-1.307	-0.589	1.923	1.00	-5.53
HETATM	8	C	MOL A	1	-6.765	-0.727	1.368	1.00	-4.52
HETATM	14	O	MOL A	1	5.693	-3.740	-0.866	1.00	0.9
HETATM	16	O	MOL A	1	8.796	-3.003	-2.410	1.00	2.05
HETATM	25	C	MOL A	1	6.500	-2.834	-2.309	1.00	5.96
HETATM	31	C	MOL A	1	9.505	-1.528	-2.459	1.00	5.96
HETATM	11	C	MOL A	1	-4.346	-1.344	-3.365	1.00	10.77

HETATM	32	C	MOL	A	1	9.613	-4.917	-0.852	1.00	14.02
HETATM	12	C	MOL	A	1	-4.014	0.983	2.255	1.00	14.27
HETATM	29	C	MOL	A	1	9.018	4.348	-2.568	1.00	14.58
HETATM	26	C	MOL	A	1	7.240	-4.458	1.485	1.00	15.14
HETATM	30	C	MOL	A	1	9.217	-3.921	2.598	1.00	16.86
HETATM	34	C	MOL	A	1	11.979	3.350	-0.662	1.00	17.23
HETATM	13	C	MOL	A	1	-3.605	-4.575	0.675	1.00	17.25
HETATM	10	C	MOL	A	1	-4.678	3.370	4.508	1.00	17.47
HETATM	28	C	MOL	A	1	8.723	-0.979	3.764	1.00	17.92
HETATM	27	C	MOL	A	1	7.638	-2.847	2.256	1.00	18.19
HETATM	7	C	MOL	A	1	-6.936	6.376	3.658	1.00	19.81
HETATM	9	C	MOL	A	1	-6.060	-6.964	1.139	1.00	20.23
HETATM	2	C	MOL	A	1	-9.764	-6.192	0.925	1.00	21.61
HETATM	33	C	MOL	A	1	11.843	0.042	1.547	1.00	21.76
HETATM	3	C	MOL	A	1	-8.114	6.852	0.011	1.00	22.46
HETATM	1	C	MOL	A	1	-10.545	-0.758	-0.549	1.00	27.09
HETATM	5	C	MOL	A	1	-7.203	2.425	-2.380	1.00	34.51

CCSQ-Tol-SCF.pdb

HETATM	11	O	MOL	A	1	1.456	4.006	-2.141	1.00	-46.97

HETATM	10	O	MOL A	1	0.931	-3.535	1.011	1.00		-45.88
HETATM	9	O	MOL A	1	-2.301	4.301	-1.650	1.00		-43.96
HETATM	8	O	MOL A	1	-2.296	-3.355	0.602	1.00		-35.02
HETATM	15	O	MOL A	1	2.518	0.443	-2.638	1.00		-32.89
HETATM	12	O	MOL A	1	2.251	0.040	1.854	1.00		-32.1
HETATM	14	O	MOL A	1	2.281	-0.909	-2.109	1.00		-31.43
HETATM	13	O	MOL A	1	2.226	1.338	1.327	1.00		-30.73
HETATM	17	O	MOL A	1	5.143	0.260	-2.283	1.00		-29.59
HETATM	16	O	MOL A	1	4.772	-0.340	1.738	1.00		-28.12
HETATM	16	C	MOL A	1	-0.530	3.958	-1.763	1.00		-25.75
HETATM	15	C	MOL A	1	-1.031	-3.133	0.746	1.00		-21.71
HETATM	20	O	MOL A	1	10.301	-1.835	-2.155	1.00		-21.69
HETATM	19	O	MOL A	1	9.953	-2.628	-1.713	1.00		-21.05
HETATM	26	C	MOL A	1	10.099	-2.322	-1.767	1.00		-20.96
HETATM	21	O	MOL A	1	11.392	-1.696	1.691	1.00		-20.08
HETATM	6	O	MOL A	1	-4.676	4.217	0.205	1.00		-16.12
HETATM	18	O	MOL A	1	8.588	-0.397	1.961	1.00		-14.28
HETATM	12	C	MOL A	1	-5.226	3.420	0.854	1.00		-12.29
HETATM	7	O	MOL A	1	-4.150	2.225	0.593	1.00		-11.58
HETATM	3	O	MOL A	1	-8.694	5.283	0.674	1.00		-5.62
HETATM	4	O	MOL A	1	-8.384	-4.913	-0.885	1.00		-5.15

HETATM	5	O	MOL A	1	-8.330	-3.528	2.916	1.00		-4.87
HETATM	1	O	MOL A	1	-9.563	-4.908	-0.863	1.00		-4.67
HETATM	2	O	MOL A	1	-9.568	-3.464	2.850	1.00		-4.4
HETATM	14	C	MOL A	1	-3.251	-0.017	1.531	1.00		0.27
HETATM	13	C	MOL A	1	-3.265	-1.232	-2.099	1.00		1.24
HETATM	22	O	MOL A	1	12.211	-5.767	-0.412	1.00		1.64
HETATM	23	O	MOL A	1	14.127	-5.288	-0.704	1.00		1.66
HETATM	23	C	MOL A	1	7.970	0.973	3.713	1.00		2.13
HETATM	17	C	MOL A	1	4.633	-3.280	-0.952	1.00		2.43
HETATM	29	C	MOL A	1	10.461	0.685	3.173	1.00		3.65
HETATM	18	C	MOL A	1	5.094	3.562	0.474	1.00		6.06
HETATM	20	C	MOL A	1	7.397	-3.434	-0.567	1.00		6.36
HETATM	19	C	MOL A	1	6.555	4.575	2.458	1.00		8.01
HETATM	22	C	MOL A	1	7.982	-3.381	1.475	1.00		8.05
HETATM	32	C	MOL A	1	12.556	-5.417	-2.288	1.00		8.82
HETATM	28	C	MOL A	1	10.234	3.136	4.389	1.00		9.28
HETATM	33	C	MOL A	1	12.911	-5.301	1.479	1.00		10.4
HETATM	24	C	MOL A	1	9.635	5.145	0.884	1.00		11.13
HETATM	21	C	MOL A	1	7.762	4.455	-0.589	1.00		11.82
HETATM	25	C	MOL A	1	9.703	3.499	-0.244	1.00		11.92

HETATM	35	C	MOL A	1	14.919	-2.524	-0.955	1.00		11.97
HETATM	27	C	MOL A	1	10.303	-5.307	1.160	1.00		12.78
HETATM	31	C	MOL A	1	11.997	2.758	-0.038	1.00		12.9
HETATM	34	C	MOL A	1	14.146	-0.794	-1.760	1.00		14.68
HETATM	30	C	MOL A	1	11.467	1.806	-1.383	1.00		17.79
HETATM	10	C	MOL A	1	-5.975	7.533	-0.444	1.00		20.1
HETATM	7	C	MOL A	1	-6.737	6.803	3.193	1.00		20.85
HETATM	8	C	MOL A	1	-6.582	-0.908	-2.373	1.00		21.11
HETATM	11	C	MOL A	1	-5.320	-4.491	1.150	1.00		22.07
HETATM	5	C	MOL A	1	-7.810	3.180	4.217	1.00		22.97
HETATM	4	C	MOL A	1	-7.850	0.735	2.202	1.00		23.84
HETATM	6	C	MOL A	1	-7.696	-6.963	2.069	1.00		23.86
HETATM	2	C	MOL A	1	-11.361	-6.452	1.826	1.00		25.4
HETATM	9	C	MOL A	1	-6.572	3.166	-3.157	1.00		32.24
HETATM	1	C	MOL A	1	-12.511	-1.080	-0.212	1.00		32.85
HETATM	3	C	MOL A	1	-9.640	1.999	-1.869	1.00		44.08

CCSQ-Tol-CI.pdb

HETATM	9	O	MOL A	1	-2.537	4.275	-1.476	1.00		-46.16
HETATM	11	O	MOL A	1	1.327	3.982	-2.282	1.00		-45.77
HETATM	10	O	MOL A	1	0.880	-3.487	1.143	1.00		-44.94

HETATM	8	O	MOL A	1	-2.541	-3.324	0.635	1.00		-38.17
HETATM	15	O	MOL A	1	2.253	0.839	-2.772	1.00		-29.63
HETATM	14	O	MOL A	1	1.978	-0.254	1.939	1.00		-28.95
HETATM	13	O	MOL A	1	2.077	-1.205	-2.058	1.00		-28.15
HETATM	12	O	MOL A	1	1.961	1.813	1.208	1.00		-27.62
HETATM	24	C	MOL A	1	-0.530	3.958	-1.763	1.00		-26.36
HETATM	19	C	MOL A	1	-1.031	-3.133	0.746	1.00		-23.03
HETATM	5	O	MOL A	1	-4.712	4.092	0.191	1.00		-21.89
HETATM	6	O	MOL A	1	-3.986	1.852	0.882	1.00		-20.96
HETATM	4	O	MOL A	1	-5.198	2.766	1.002	1.00		-20.22
HETATM	15	C	MOL A	1	-5.212	3.554	0.862	1.00		-19.2
HETATM	7	O	MOL A	1	-3.471	0.109	-2.704	1.00		-16.73
HETATM	22	C	MOL A	1	-0.682	2.244	0.351	1.00		-15.9
HETATM	23	C	MOL A	1	-0.563	1.310	-2.445	1.00		-15.79
HETATM	21	C	MOL A	1	-0.762	-1.464	-1.503	1.00		-14.14
HETATM	20	C	MOL A	1	-0.925	-0.518	1.309	1.00		-14.05
HETATM	19	O	MOL A	1	10.499	-1.923	-2.226	1.00		-13.38
HETATM	18	O	MOL A	1	10.050	-2.770	-1.740	1.00		-12.75
HETATM	18	C	MOL A	1	-2.126	0.399	-2.674	1.00		-12.4
HETATM	20	O	MOL A	1	11.559	-1.788	1.673	1.00		-12.15

HETATM	2	O	MOL	A	1	-8.262	-4.917	-0.902	1.00	-11.1
HETATM	1	O	MOL	A	1	-8.726	5.172	0.762	1.00	-10.9
HETATM	3	O	MOL	A	1	-8.347	-3.436	2.866	1.00	-10.81
HETATM	17	C	MOL	A	1	-3.212	-0.277	1.797	1.00	-9.4
HETATM	16	C	MOL	A	1	-3.219	-1.605	-2.144	1.00	-8.78
HETATM	4	C	MOL	A	1	-9.053	-3.986	-1.025	1.00	-7.87
HETATM	5	C	MOL	A	1	-9.014	-2.651	2.341	1.00	-7.51
HETATM	16	O	MOL	A	1	8.588	-0.397	1.961	1.00	-3.73
HETATM	17	O	MOL	A	1	9.251	4.645	3.272	1.00	6.25
HETATM	21	O	MOL	A	1	14.127	-5.288	-0.704	1.00	6.26
HETATM	22	O	MOL	A	1	14.265	-4.473	-1.865	1.00	6.38
HETATM	11	C	MOL	A	1	-6.596	-1.044	-2.387	1.00	8.26
HETATM	31	C	MOL	A	1	8.079	0.806	3.552	1.00	8.89
HETATM	25	C	MOL	A	1	4.895	-3.286	-0.955	1.00	8.9
HETATM	12	C	MOL	A	1	-6.414	-1.867	-3.108	1.00	9.07
HETATM	36	C	MOL	A	1	10.458	0.570	3.128	1.00	10.63
HETATM	26	C	MOL	A	1	5.255	3.540	0.476	1.00	12.51
HETATM	27	C	MOL	A	1	6.597	4.611	2.339	1.00	13.2
HETATM	38	C	MOL	A	1	12.522	-5.348	-2.344	1.00	13.58
HETATM	35	C	MOL	A	1	10.236	3.004	4.391	1.00	14.35
HETATM	28	C	MOL	A	1	7.397	-3.434	-0.567	1.00	14.6

HETATM	39	C	MOL	A	1	12.809	-5.301	1.471	1.00	15.24
HETATM	14	C	MOL	A	1	-5.399	-4.639	1.165	1.00	15.71
HETATM	29	C	MOL	A	1	7.874	-3.334	1.403	1.00	15.71
HETATM	6	C	MOL	A	1	-7.966	0.919	2.341	1.00	15.93
HETATM	13	C	MOL	A	1	-5.975	7.533	-0.444	1.00	16.54
HETATM	32	C	MOL	A	1	9.641	5.036	0.738	1.00	16.67
HETATM	41	C	MOL	A	1	14.714	-2.338	-1.076	1.00	17.09
HETATM	9	C	MOL	A	1	-6.737	6.803	3.193	1.00	17.28
HETATM	8	C	MOL	A	1	-7.725	3.285	4.266	1.00	18.19
HETATM	30	C	MOL	A	1	7.923	4.382	-0.697	1.00	18.44
HETATM	34	C	MOL	A	1	10.303	-5.307	1.160	1.00	18.45
HETATM	7	C	MOL	A	1	-7.696	-6.963	2.069	1.00	19.76
HETATM	33	C	MOL	A	1	9.687	3.484	-0.312	1.00	19.78
HETATM	40	C	MOL	A	1	14.146	-0.794	-1.760	1.00	20.41
HETATM	2	C	MOL	A	1	-11.361	-6.452	1.826	1.00	21.52
HETATM	10	C	MOL	A	1	-6.628	3.287	-3.066	1.00	24.62
HETATM	37	C	MOL	A	1	11.353	1.843	-1.369	1.00	25.59
HETATM	1	C	MOL	A	1	-12.511	-1.080	-0.212	1.00	27.76
HETATM	3	C	MOL	A	1	-9.681	1.919	-1.899	1.00	36.46

CCSQ-Tol-CIOPT.pdb

HETATM	9	O	MOL	A	1	-2.557	4.320	-1.287	1.00	-48.85
HETATM	11	O	MOL	A	1	1.279	4.175	-1.948	1.00	-47.66
HETATM	10	O	MOL	A	1	0.860	-3.666	0.682	1.00	-46.83
HETATM	8	O	MOL	A	1	-2.532	-3.366	0.471	1.00	-40.58
HETATM	15	O	MOL	A	1	2.227	0.888	-2.713	1.00	-31.2
HETATM	12	O	MOL	A	1	1.904	-0.333	1.833	1.00	-30.59
HETATM	14	O	MOL	A	1	1.978	-1.223	-2.164	1.00	-30.27
HETATM	13	O	MOL	A	1	1.937	1.762	1.284	1.00	-29.8
HETATM	23	C	MOL	A	1	-0.519	4.043	-1.500	1.00	-29.56
HETATM	18	C	MOL	A	1	-0.948	-3.232	0.452	1.00	-26.21
HETATM	5	O	MOL	A	1	-4.816	4.196	0.290	1.00	-23.04
HETATM	6	O	MOL	A	1	-3.905	1.792	0.952	1.00	-22.27
HETATM	4	O	MOL	A	1	-5.159	3.020	1.251	1.00	-21.72
HETATM	15	C	MOL	A	1	-5.196	3.654	0.830	1.00	-20.57
HETATM	22	C	MOL	A	1	-0.672	2.210	0.474	1.00	-19.15
HETATM	21	C	MOL	A	1	-0.597	1.389	-2.380	1.00	-18.89
HETATM	7	O	MOL	A	1	-3.390	0.195	-2.670	1.00	-18.29
HETATM	20	C	MOL	A	1	-0.719	-1.400	-1.606	1.00	-17.39
HETATM	19	C	MOL	A	1	-0.824	-0.705	1.244	1.00	-16.99
HETATM	17	C	MOL	A	1	-2.180	0.466	-2.641	1.00	-15.01
HETATM	2	O	MOL	A	1	-8.387	-4.822	-1.029	1.00	-12.11

HETATM	3	O	MOL A	1	-8.270	-3.514	2.798	1.00		-11.87
HETATM	1	O	MOL A	1	-8.846	4.870	1.154	1.00		-11.71
HETATM	19	O	MOL A	1	10.530	-2.176	-2.089	1.00		-10.69
HETATM	16	C	MOL A	1	-3.330	-0.439	2.038	1.00		-10.53
HETATM	18	O	MOL A	1	10.072	-3.001	-1.492	1.00		-10.08
HETATM	20	O	MOL A	1	11.619	-1.630	1.773	1.00		-9.45
HETATM	4	C	MOL A	1	-9.013	-3.966	-1.099	1.00		-8.99
HETATM	5	C	MOL A	1	-9.031	-2.694	2.296	1.00		-8.68
HETATM	16	O	MOL A	1	8.446	-0.180	1.880	1.00		1.28
HETATM	11	C	MOL A	1	-6.685	-0.989	-2.367	1.00		7.3
HETATM	17	O	MOL A	1	9.365	4.874	2.845	1.00		7.61
HETATM	21	O	MOL A	1	13.984	-5.485	-0.098	1.00		7.67
HETATM	22	O	MOL A	1	14.240	-4.744	-1.348	1.00		7.84
HETATM	12	C	MOL A	1	-6.444	-1.782	-3.162	1.00		8.15
HETATM	24	C	MOL A	1	5.112	-3.297	-0.794	1.00		10.16
HETATM	30	C	MOL A	1	8.294	1.146	3.355	1.00		10.88
HETATM	32	C	MOL A	1	10.142	0.619	3.062	1.00		12.86
HETATM	25	C	MOL A	1	5.429	3.566	0.188	1.00		13.78
HETATM	26	C	MOL A	1	6.710	4.857	1.891	1.00		14.34
HETATM	7	C	MOL A	1	-7.581	0.624	2.269	1.00		14.87

HETATM	14	C	MOL	A	1	-5.421	-4.688	1.007	1.00	14.99
HETATM	36	C	MOL	A	1	12.480	-5.682	-1.700	1.00	15.05
HETATM	34	C	MOL	A	1	10.232	3.354	4.124	1.00	15.76
HETATM	27	C	MOL	A	1	7.387	-3.464	-0.477	1.00	16.09
HETATM	13	C	MOL	A	1	-6.290	7.573	-0.381	1.00	16.28
HETATM	10	C	MOL	A	1	-6.773	6.733	3.382	1.00	16.51
HETATM	37	C	MOL	A	1	12.630	-5.139	2.015	1.00	16.83
HETATM	8	C	MOL	A	1	-7.525	3.137	4.396	1.00	17.02
HETATM	28	C	MOL	A	1	7.851	-3.119	1.745	1.00	17.35
HETATM	6	C	MOL	A	1	-7.721	-7.024	1.882	1.00	18.96
HETATM	29	C	MOL	A	1	8.124	4.236	-1.160	1.00	19.96
HETATM	33	C	MOL	A	1	10.249	-5.133	1.750	1.00	20.16
HETATM	2	C	MOL	A	1	-11.465	-6.415	1.719	1.00	20.78
HETATM	31	C	MOL	A	1	9.791	3.431	-0.604	1.00	21.97
HETATM	38	C	MOL	A	1	14.162	-1.063	-1.689	1.00	22.34
HETATM	9	C	MOL	A	1	-6.927	3.443	-2.880	1.00	23.98
HETATM	1	C	MOL	A	1	-12.534	-1.129	-0.134	1.00	26.75
HETATM	35	C	MOL	A	1	11.345	1.678	-1.548	1.00	28.28
HETATM	3	C	MOL	A	1	-9.664	2.059	-1.780	1.00	35.27
